# A disordered region retains the full protease inhibitor activity and the capacity to induce CD8^+^ T cells *in vivo* of the oral vaccine adjuvant U-Omp19

**DOI:** 10.1016/j.csbj.2022.08.054

**Published:** 2022-09-06

**Authors:** M. Laura Darriba, Celeste Pueblas Castro, Lorena M. Coria, Laura Bruno, M. Laura Cerutti, Lisandro H. Otero, Lucía B. Chemes, Rodolfo M. Rasia, Sebastián Klinke, Juliana Cassataro, Karina A. Pasquevich

**Affiliations:** a Instituto de Investigaciones Biotecnológicas, Universidad Nacional de San Martín (UNSAM) – Consejo Nacional de Investigaciones Científicas y Técnicas (CONICET), Buenos Aires, Argentina; Escuela de Bio y Nanotecnologías (EByN), Universidad Nacional de San Martín, Buenos Aires, Argentina; c Fundación Instituto Leloir, IIBBA-CONICET, and Plataforma Argentina de Biología Estructural y Metabolómica PLABEM, Buenos Aires, Argentina; d Instituto de Biología Molecular y Celular de Rosario (IBR-CONICET), Facultad de Ciencias Bioquímicas y Farmacéuticas, Universidad Nacional de Rosario, Santa Fe, Argentina and Plataforma Argentina de Biología Estructural y Metabolómica PLABEM, Buenos Aires, Argentina

**Keywords:** Mucosal adjuvant, Protease inhibitor, Structure-activity relationship, Protein structure, Protein crystallization, U-Omp19, unlipidated outer membrane protein 19, IMAC, immobilized metal ion affinity chromatography, SEC, size exclusion chromatography, TEV, Tobacco Etch Virus, CD, Circular dichroism, SLS, Static light scattering, GdmCl, guanidinium chloride, MW, molecular weight, IDP, intrinsically disordered protein, NMR, nuclear magnetic resonance, MBP, maltose binding protein

## Abstract

U-Omp19 is a bacterial protease inhibitor from *Brucella abortus* that inhibits gastrointestinal and lysosomal proteases, enhancing the half-life and immunogenicity of co-delivered antigens. U-Omp19 is a novel adjuvant that is in preclinical development with various vaccine candidates. However, the molecular mechanisms by which it exerts these functions and the structural elements responsible for these activities remain unknown. In this work, a structural, biochemical, and functional characterization of U-Omp19 is presented. Dynamic features of U-Omp19 in solution by NMR and the crystal structure of its C-terminal domain are described. The protein consists of a compact C-terminal beta-barrel domain and a flexible N-terminal domain. The latter domain behaves as an intrinsically disordered protein and retains the full protease inhibitor activity against pancreatic elastase, papain and pepsin. This domain also retains the capacity to induce CD8^+^ T cells *in vivo* of U-Omp19. This information may lead to future rationale vaccine designs using U-Omp19 as an adjuvant to deliver other proteins or peptides in oral formulations against infectious diseases, as well as to design strategies to incorporate modifications in its structure that may improve its adjuvanticity.

## Graphical abstract

**Figure:**
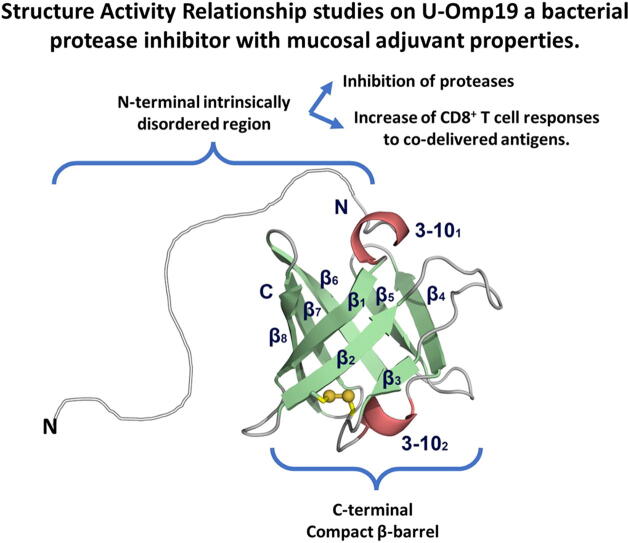

## 1 Introduction

A major goal in vaccine design is to induce protective and lasting immunity against pathogens at mucosal surfaces. While most currently available vaccines are administered parenterally, this method usually fails to elicit immune responses at mucosal sites which are the main portal of entry of pathogens. Conversely, mucosal immunization can induce strong protective immunity not only at the mucosa but also systemically. Besides, oral vaccines offer needle-free delivery, improved accessibility, safety, cost-effectiveness and may be the preferred choice for mass vaccination. However, oral delivery is challenging, requiring formulations to overcome the harsh proteolytic gastrointestinal environment and avoid tolerance induction to achieve effective protection. These could be achieved by the addition of mucosal adjuvants [1]. Adjuvants are vaccine components that enhance the magnitude, breadth, and durability of the immune response. Although adjuvants provide a rational and attractive tool for vaccine design, only a few are currently included in licensed vaccines [2], [3] and these are lacking for oral vaccines [4].

Previous work of our laboratory has shown that the unlipidated outer membrane protein 19 (U-Omp19) from *Brucella abortus* can be used as an adjuvant in oral or parenteral vaccine formulations [5], [6], [7], [8], [9], [10], [11], [12], [13]. The addition of U-Omp19 to oral vaccine formulations enhances vaccine induced protection against *Escherichia coli* or *Vibrio cholerae* toxin-induced diarrhea or oral challenge with *Salmonella*, Enterotoxigenic *E. coli*, Enterohemorrhagic *E. coli* O157:H7 or *Toxoplasma gondii* in mice [5], [6], [7]. Also, the addition of U-Omp19 to a SARS-CoV-2 intramuscular subunit vaccine enhanced the induced neutralizing antibody response and elicited protection against SARS-CoV-2 challenge [14].

*In silico* studies have predicted that U-Omp19 is structurally related to the Inh domain [7], a characteristic β-barrel present in proteins belonging to the I38 family of bacterial protease inhibitors [15]. Previous work has shown that U-Omp19 is a protease inhibitor that partially inhibits gastrointestinal and lysosomal proteases [7], [9]. This activity allows U-Omp19 to protect co-delivered antigens (Ags) from gastrointestinal and intracellular proteases increasing their half-life and immunogenicity [5], [6], [7], [8], [9].

Protease inhibitors are ubiquitous regulatory proteins that reduce the activity of target proteases. Specific protease inhibitors are effective tools for inactivating proteases involved in human diseases like arthritis, pancreatitis, hepatitis, cancer, AIDS, thrombosis, etc. [16]. To our knowledge there are no other protease inhibitors used as adjuvants. Therefore, U-Omp19 represents a new concept in vaccine adjuvant development.

Structure-activity relationship knowledge of U-Omp19 will allow a better understanding of the molecular mechanisms involved in its protease inhibitor activity and vaccine adjuvant properties. Besides it will shed light on the role of Omp19 in the context of the *Brucella* infection [17].

To understand the mechanistic basis of U-Omp19 activities, in this work we performed a structural, biochemical, and functional characterization of it. These analyses shed light on the structure and function of key regions of the molecule involved in inhibition of proteases and adjuvant activity. Collectively, the results of this work may contribute to the understanding of the mechanisms of action of U-Omp19 and will enable the design of novel engineered structures that may have enhanced activity, stability, or the conjugation to other bioactive molecules or Ags.

## 2 Materials and methods

### 2.1 Cloning

Protein constructs used in present work are listed in Table 1.

**Table 1:** Protein constructs used in the present work.

Construct name	Domain architecture	MW_THEO_ (kDa)^a^	N of residues	Purpose
U-Omp19	Full length U-Omp19: U-Omp19 (1–159) - (His)_6_	16.8	165	U-Omp19 characterization experiments
MBP-U-Omp19	MBP – AAAM - U-Omp19 (57–159) - (His)_6_	51.7	481	Crystallization experiments
U-Omp19-X-TEV (9 different constructs)	(His)_6_ - U-Omp19 – X - TEV Where X is the number of the residue where a TEV heptapeptide cleavage site (ENLYFQ/G) was added. (X = 1, 5, 10, 15, 20, 32, 40, 53, 60)	17.5	170	Expression of truncated fragments of U-Omp19 for mapping the active region
U-Omp19_(2-159)_	G - U-Omp19 (2–159)	15.7	157	Mapping active regions (A)
U-Omp19_(5-159)_	G - U-Omp19 (5–159)	15.4	154	(A)
U-Omp19_(10-159)_	G - U-Omp19 (10–159)	14.9	149	(A)
U-Omp19_(15-159)_	G - U-Omp19 (15–159)	14.4	144	(A)
U-Omp19_(20-159)_	G - U-Omp19 (20–159)	13.9	139	(A)
U-Omp19_(32-159)_	U-Omp19 (32–159)	12.7	126	(A)
U-Omp19_(40-159)_	G - U-Omp19 (40–159)	11.9	119	(A)
U-Omp19_(53-159)_	U-Omp19 (53–159)	10.5	105	(A)
U-Omp19_(60-159)_	G - U-Omp19 (60–159)	9.9	99	(A) and NMR experiments.
U-Omp9_(1–60)_	U-Omp19 (1–60) - ENLYFQ derived from the TEV cleavage site.	6.8	66	(A)

^a^ Theoretical MW computed by the ExPASy ProtParam server (22).

#### 2.1.1 U-Omp19 characterization experiments

The U-Omp19 sequence was cloned in the pET22b+ vector (Novagen, Madison, WI) resulting in the pET-U-Omp19 plasmid as previously described [7], [18].

#### 2.1.2 Crystallization experiments

For crystallization experiments, the predicted compact domain of U-Omp19 (residues 57 to 159) plus the histidine tag (residues 160 to 165) was C-terminally fused to maltose binding protein (MBP) containing mutations designed to reduce surface entropy and encourage crystal lattice formation [19]. The plasmid was generated by standard recombinant cloning techniques and the final construct was verified by DNA sequencing. Briefly, the sequence corresponding to the U-Omp19 C-terminal region (residues 57–165) was amplified by PCR using pET-U-Omp19 as template. 5′-CCATGGCAAGCCTGCCGCCTGCATCC-3′ was used as forward primer, and 5′-GGTACCGCAGCCGGATCTCAGTGGTGG-3′ as reverse primer. *Nco*I or *Kpn*I cleavage sites were added to each oligonucleotide respectively to be further annealed and cloned into the digested pMALc-2 vector, giving rise to the pMALc-2-U-Omp19 vector. The final engineered protein consisted in the fusion of MBP to the C-terminal domain of U-Omp19 linked by 3 alanine and 1 methionine residues (AAAM) and a C-terminal 6 × -His tag.

#### 2.1.3 Truncated proteins of U-Omp19

For the obtention of the different C-terminal fragments of U-Omp19, first, pET-(His)_6_-U-Omp19 was generated and used as template for the addition of the Tobacco Etch Virus (TEV) protease cleavage site at diverse sequence points (Table 1) using the mutagenesis service of Genscript (Piscataway, NJ, USA). For the obtention of the U-Omp19 N-terminal fragment, the pET-U-Omp19 plasmid was used as template and the TEV protease cleavage site was inserted after the residue Pro60 of the full-length protein.

### 2.2 Protein purification

#### 2.2.1 U-Omp19 characterization experiments

Recombinant U-Omp19 was expressed in *E. coli* BL21(DE3) and purified by immobilized metal ion affinity chromatography (IMAC) and size exclusion chromatography (SEC) as described previously [18].

#### 2.2.2 Crystallization experiments

*E. coli* BL21(DE3) cells were transformed with the pMALc-2-U-Omp19 plasmid, grown in antibiotic containing medium and protein expression was induced with addition of 1 mM isopropyl-β-d-thiogalactopyranoside (IPTG). Cells were harvested and disrupted by sonication. The protein was purified from the supernatant by FPLC using a HisTrap HP column. Eluates were concentrated by centrifugation in Amicon Ultra centrifugal filters (10,000 Da MW cut-off) and further purified by SEC using a 16/60 Superdex 75 column. The final samples were concentrated to 34 mg/ml in Amicon filters and simultaneously exchanged into low ionic strength crystallization buffer (10 mM Tris-HCl, 25 mM sodium chloride, pH 7.5).

#### 2.2.3 Truncated proteins of U-Omp19

The full-length U-Omp19-X-TEV constructs were expressed and purified by FPLC using a HisTrap HP column. A series of truncated variants of U-Omp19 were generated by site-specific cleavage of the full-length protein by TEV protease (Table 1). Purified proteins were buffer exchanged to TEV cleavage buffer [20] and mixed with His-tagged TEV protease [20] at 10:1 M ratio for 2 days at 18 °C. After digestion, digested proteins were further purified in a second HisTrap step to capture the remaining non-cleaved protein, TEV protease and the released 6 × -His-tagged fragments and then further purified by SEC on a 16/60 Superdex-75 column.

#### 2.2.4 NMR analysis

^15^N or ^13^C, ^15^N-uniformly labeled full-length U-Omp19 or U-Omp19_(60-159)_ were obtained by growing of *E. coli* BL21(DE3) cells transformed with respective expression plasmids (Table 1) in M9 minimal medium supplemented with 1 g/l [^15^N] ammonium chloride with or without 2 g/l [U-^13^C] glucose (Cambridge Isotope Laboratories), respectively. Expression and purification protocols were performed as described previously for each protein construct.

#### 2.2.5 *In vivo* adjuvanticity experiments

After being purified as previously described in each section, for the *in vivo* analysis, proteins were depleted of lipopolysaccharide (LPS) using a polymyxin B resin (Sigma). Endotoxin determinations were performed with a Limulus amebocyte chromogenic assay (Lonza). Proteins used for *in vivo* assays contained less than 0.1 endotoxic units (EU)/mg.

#### 2.2.6 Protein purity and concentration determination

The quality of all final purified protein samples was checked by SDS-PAGE (15 % gel) and UV–vis spectrophotometry. The protein concentration was estimated by measuring its absorbance at λ = 280 nm. The theoretical molar extinction coefficient of each purified protein was estimated from its sequence using the ProtParam tool from the ExPASy server [21].

### 2.3 SDS-PAGE and Western blotting (WB)

U-Omp19 samples were run under both non-reducing and reducing conditions by SDS-PAGE as described previously [18]. For WB, after being subjected to SDS-PAGE, samples were transferred onto a nitrocellulose membrane. A polyclonal rabbit anti-U-Omp19 serum followed by anti-rabbit IgG labeled with IRDye 680 (LI-COR Biosciences) were used to visualize the bands. Images were captured and documented with an Odyssey infrared image-scanner (LI-COR Biosciences).

### 2.4 Circular dichroism (CD)

CD measurements were performed as previously described [18]. Samples were diluted to 10 μM or 100 μM for Far- or Near-UV CD experiments respectively. Raw data were then converted to molar ellipticity using the following equation:

$$\left[\theta \right]MRW\left(deg.{\mathrm{cm}}^{2}.{\mathrm{dmol}}^{-1}\right)=\frac{\theta }{\#bonds\left[C\right]10L}$$

where θ is the raw CD signal in millidegrees, [C] is the molar protein concentration, #bonds is the number of peptide bonds, and L is the path length in centimeters. The temperature was 20 °C unless otherwise stated. The deconvolution of the CD spectra were performed using the BeStSel server [22].

For thermal denaturation experiments, the ellipticity at 230 nm was recorded as the temperature was increased or decreased at a scan rate of 5 °C/min from 20 to 100 °C. Acquisition parameters were as follows: 1 nm bandwidth, 2 s response time, and 0.1 nm data pitch. The concentration of U-Omp19 was set to 20 μM. Thermal unfolding curves were fit to a two-state model where the unfolding free energy (ΔG(T)) can be calculated as a function of temperature (T) as follows:

$$\Delta G(T)=\frac{\mathrm{Tm}-T}{\mathrm{Tm}}+\Delta H(\mathrm{Tm})+\Delta Cp\times \left[T-\mathrm{Tm}-T\times \ln\frac{T}{\mathrm{Tm}}\right]$$

Here, Tm is the midpoint temperature, ΔH(Tm) is the enthalpy change of unfolding at Tm and ΔCp is the change in heat capacity between the folded and unfolded states. This last parameter was estimated by calculating the change in solvent accessible surface area (SASA) between the folded and unfolded states [23] and kept fixed during the data fitting procedure. The change in SASA was calculated using the program ProtSA [24]. The experimental data were fitted using the curve fit function of the SciPy package, assuming a linear dependence of ellipticity with temperature in the pre- and post-transition regions, and fitting errors were estimated as the square root of the covariance matrix diagonal elements.

Chemical denaturation was evaluated by Far-UV CD. For this purpose, a concentrated stock of U-Omp19 (in 10 mM NaH_2_PO_4_, pH 7.0) was diluted with a solution of 10 M urea or 6 M GdmCl in the same buffer until a final protein concentration of 10 μM and a series of final concentrations of denaturing agent. After 24 h at room temperature, the entire CD spectrum in the Far-UV region was acquired as detailed above. To evaluate the cooperative effect, the ellipticity at 218 nm was plotted as a function of the concentration of the denaturing agent. The reversibility of the structural transitions was assessed by monitoring the recovery of the CD spectra after (i) the heated protein was returned to room temperature and (ii) the protein in 9 M urea or 5 M GdmCl was diluted to the lowest concentration of the chemical agent. The spectra measured in the presence of DTT, GdmCl or Urea were reliable between 210 and 260 nm.

Urea unfolding and refolding curves were obtained from Far-UV CD spectra obtained at different denaturant concentrations that were decomposed applying singular value decomposition (SVD) to the full matrix of spectra. The population of the main components was then used to plot the fraction of unfolded protein versus denaturant concentration.

### 2.5 Static light scattering (SLS)

The average MW of U-Omp19 was determined by static light scattering (SLS) on a Precision Detectors PD2010 90° light scattering instrument tandemly connected to an HPLC apparatus, including a Waters 486 UV detector and an LKB 2142 differential refractometer. The chromatographic runs were performed in a Superdex 75 GL 10/300 column. The elution was monitored by measuring its SLS signal at 90°, its UV absorption at λ = 280 nm, and its refractive index (RI). Data were analyzed with the Discovery32 software supplied by Precision Detectors. The MW of each sample was calculated relating its SLS and RI signals and comparing this value with the one obtained for Bovine serum albumin (MW 66.5 kDa).

### 2.6 Size exclusion chromatography (SEC)

Samples were analyzed by isocratic SEC on an analytical Superdex 75 10/300 GL column. The void (V_0_) and total (V_T_) volume were determined by loading Blue Dextran and acetone, respectively. All runs were performed in 50 mM sodium phosphate, 200 mM sodium chloride, pH 7.4 buffer. The flow rate was 0.4 ml/min, and the detection was set at λ = 280 nm. The protein mass loaded was 200 µg with an injection volume of 250 μl.

### 2.7 Hydrodynamic behavior analysis

To analyze the hydrodynamic properties of the different U-Omp19 fragments, the apparent MW was calculated using the elution volumes obtained by SEC (MW_SEC_). Briefly, elution volumes (V_e_) of globular protein standards were used to estimate partition coefficients as K_av_ = (V_e_ − V_0_)/(V_T_ − V_0_). The MW of each protein was then estimated by interpolation using a calibration curve obtained as the linear regression of K_av_ versus log (MW) of the standards (Goodness of fit R square = 0.9576). The deviation from the hydrodynamic behavior expected for a globular protein was analyzed by calculating the ratio MW_SEC_/MW_THEO_ (where MW_THEO_ is the theoretical MW computed by the ExPASy ProtParam server (Table 1)). For a typical monomeric, globular, ideally spherical proteins, the expected MW_SEC_/MW_THEO_ ratio is close to 1, whereas for extended structures and denatured proteins, ratios should be significantly higher [25].

### 2.8 Crystallization, X-ray data collection and structure resolution of MBP-U-Omp19

An initial screening of crystallization conditions was performed at room temperature on 96-well plates in a sitting drop vapor diffusion configuration using a Honeybee 963 robot (Digilab, Marlborough, MA, USA) and commercial kits from Jena Bioscience (Jena, Germany) and Hampton Research (Aliso Viejo, CA, USA). Experiments were carried out in duplicates with or without the addition of 10 mM maltose. After 4 days of equilibration, crystals appeared in two maltose-free conditions: (i) 2.0 M ammonium sulfate (tiny bars) and (ii) 2.4 M ammonium sulfate, 0.1 M sodium citrate (bipyramids). Crystals were optimized by the hanging drop method by mixing 1:1 the protein stock with the crystallization solutions. Samples were cryoprotected in mother liquor supplemented with 35 % (*v*/*v*) glycerol and flash-cooled in liquid nitrogen using Hampton Research loops.

X-ray diffraction data were collected on several crystals at 100 K at the PROXIMA-1 protein crystallography beamline at the SOLEIL Synchrotron (France) with a PILATUS 6 M detector (Dectris, Baden, Switzerland). Data sets were indexed, integrated and scaled with XDS [26] and AIMLESS [27]. A total of 5 % of the recorded reflections were flagged for cross validation. The best diffracting crystal corresponded to a sample grown in condition (ii), yielding a complete data set at 2.55 Å resolution in the tetragonal space group *I*4_1_22 with excellent statistics.

The MBP-U-Omp19 structure was solved by the molecular replacement method with PHENIX [28] using Phaser-MR. For this purpose, MBP (PDB code 1LLS) was selected as search model. The top solution was subjected to an initial refinement step in phenix.refine, yielding *R* = 0.324 and *R*_free_ = 0.349, followed by an automated model building step in AutoBuild [29] (*R* = 0.227, *R*_free_ = 0.257). At this point, most of the U-Omp19 fragment was successfully traced. Further refinement and manual model building were then performed with phenix.refine and Coot [30], respectively. The final model was validated with MolProbity [31]. Detailed statistics on the data collection and refinement steps are shown in Table 2.

**Table 2:** X-ray diffraction data collection and refinement statistics.

*Data collection*	
Wavelength (Å)	0.9763
Crystal-detector distance (mm)	557.20
Rotation range per image (°)	0.1
No. of frames	1800
Exposure time per image (s)	0.1
*Indexing and scaling*	
Cell parameters	
a = b (Å)	141.67
c (Å)	132.22
α = β = γ (°)	90
Space group	*I*4_1_22
Mosaicity (°)	0.075
Resolution range (Å)	48.33 – 2.55
Total No. of reflections	286,075
No. of unique reflections	22,227
Completeness (%) ^a^	100.0 (100.0)
Redundancy	12.9 (11.1)
〈I/σ(I)〉	16.2 (1.3)
*R*_meas_	0.108 (1.928)
*R*_pim_	0.030 (0.575)
CC_1/2_ (%)	99.9 (55.0)
Solvent content (%)	62
No. of chains per asymmetric unit	1
Overall *B-*factor from Wilson plot (Å^2^)	59
*Refinement*	
Number of protein atoms	3575
Number of ligand atoms	35
Number of water molecules	37
*R*	0.207
*R*_free_	0.242
Rms deviations from ideal values [74]	
Bond lengths (Å)	0.003
Bond angles (°)	0.639
Average *B-*factor (Å^2^)	68
*MolProbity validation*[32]	
Clashscore (percentile)	4.90 (99th)
MolProbity score (percentile)	1.57 (99th)
Ramachandran plot	
Favored (%)	95.5
Allowed (%)	4.3
Disallowed (%)	0.2
*Protein Data Bank deposition*	
PDB code	7MHW

^a^Values for the outer shell are given in parentheses (2.66 – 2.55 Å).

Superpositions and rmsd calculations were done with the PDBeFold server at the European Bioinformatics Institute (EBI, https://www.ebi.ac.uk/msd-srv/ssm/). The study of interfaces, monomers and assemblies was done with the PDBePISA server at the EBI (https://www.ebi.ac.uk/msd-srv/prot_int/).

### 2.9 NMR spectroscopy

NMR data were collected on a 700 MHz Bruker spectrometer equipped with an Avance III console and a TXI-Z probe at 298 K. All spectra were processed with NMRPipe [32] and analyzed with CCP NMR v.2 [33]. Chemical shifts were referenced with respect to the H_2_O signal at 4.77 ppm (pH 6.8, 25 °C) relative to DSS, using the 1H:X frequency ratios of the zero point [34]. Backbone ^1^H, ^13^C, and ^15^N chemical shifts of U-Omp19 were assigned using a set of triple-resonance spectra [BEST-HNCA/HN(CO)CA, BEST-HNCACB/HN(CO)-CACB, BEST-HN(CA)CO/HNCO, and HN(CA)HA] [35]. The probability of secondary structure element distributions based on backbone chemical shift were calculated using the δ2d server [36].

^15^N relaxation datasets (T1, T2 and ^1^H-^15^N NOE) were acquired at 700 MHz using standard experiments from the Bruker library. The parameters were calculated in CCP NMR v 2 and the data were analyzed within the model-free formalism [37], [38] using the program FAST-Modelfree [39].

### 2.10 Graphical representation

Molecular structures were represented using PyMOL (Schrödinger, USA) and UCSF ChimeraX [40].

### 2.11 Protease inhibitor activity

The inhibitory activities of U-Omp19 and truncated variants were screened against pancreatic elastase, papain and pepsin as previously described [7], [9], [18]. Percentage of residual protease activity was calculated as the percentage of residual protease activity when the inhibitor is added compared to “No inhibitor” condition (100 % of protease activity). Inhibitor activity was calculated as the difference of 100 % minus the percentage of residual protease activity for the protein tested. To compare the inhibitor activity from different proteins and to normalize data, the inhibitor activity ratio was calculated as the ratio of inhibitor activity of the protein tested and inhibitor activity of full-length U-Omp19. A ratio = 1 indicates retention of full inhibitory activity, while a ratio < 1 indicates loss of inhibitory activity.

### 2.12 *In vivo* adjuvanticity experiments

#### 2.12.1 Ethics statement

All experimental protocols with animals were conducted in strict accordance with international ethical standards for animal experimentation (Declaration of Helsinki and its amendments, Amsterdam protocol of welfare and animal protection, and National Institutes of Health Guide for the Care and Use of Laboratory Animals). Protocols of this work were approved by the Institutional Committee for the Care and Use of Laboratory Animals from the University of San Martin (UNSAM, Buenos Aires, Argentina) (CICUAE N° 15/2017).

#### 2.12.2 Animals

Eight-week-old female C57BL/6 mice were obtained and housed in our local animal facility at IIB-UNSAM. OT-I/RAG1 (OT-I) mice were obtained from The Jackson Laboratory and were bred in the animal facility of IIB-UNSAM.

#### 2.12.3 Adoptive transfer of OT-I cells and *in vivo* CD8^+^ T cell proliferation

Single-cell suspensions of spleen and lymph node cells from OT-I mice were labeled with CFSE (Molecular Probes) and injected i.v. in C57BL/6 sex-matched recipients. Transferred mice were then immunized by gavage with a single dose of OVA (5 mg); OVA (5 mg) plus U-Omp19 (1 mg = 60 μmole); OVA (5 mg) plus U-Omp19_(60-159)_ (60 μmole); OVA (5 mg) plus U-Omp19_(1–60)_ (60 μmole), or saline. Three days after immunization, mice were sacrificed, and spleen cell suspensions were obtained to evaluate the proliferation of CD8^+^ T cells by flow cytometry. Cells were acquired on a BD Fortessa X-20 flow cytometer (BD Bioscience) and analyzed using the FlowJo X software (FlowJo, LLC).

### 2.13 Statistical analysis

Statistical analysis and plotting were performed using GraphPad Prism 7 (GraphPad Software, San Diego, CA). In all graphs, when bars are plotted, results are expressed as mean ± SEM. For the estimation of the MW_SEC_, the samples were analyzed in triplicate. The standard curve was performed as indicated on item 7 of this section. For comparing secondary structure content, data were analyzed using two-way ANOVA with a Bonferroni’s multiple comparisons test. For the protease inhibitor activity calculation, tests were carried out in duplicate in at least three independent experiments for each sample. The results obtained were analyzed with one-way ANOVA followed by Bonferroni’s test *vs* “No inhibitor” condition or U-Omp19 inhibitory activity. For *in-vivo* experiments, data represent pooled results of four independent experiments with n = 2–4/group for each trial (mean ± SEM), results were then analyzed using one-way ANOVA followed by Bonferroni’s test *vs* OVA or OVA + U-Omp19 condition.

## 3 Results

### 3.1 *In silico* studies and biophysical characterization of U-Omp19 reveal the presence of an extended disordered N-terminal region and a C-terminal domain rich in β-structure

**Fig. 1:**
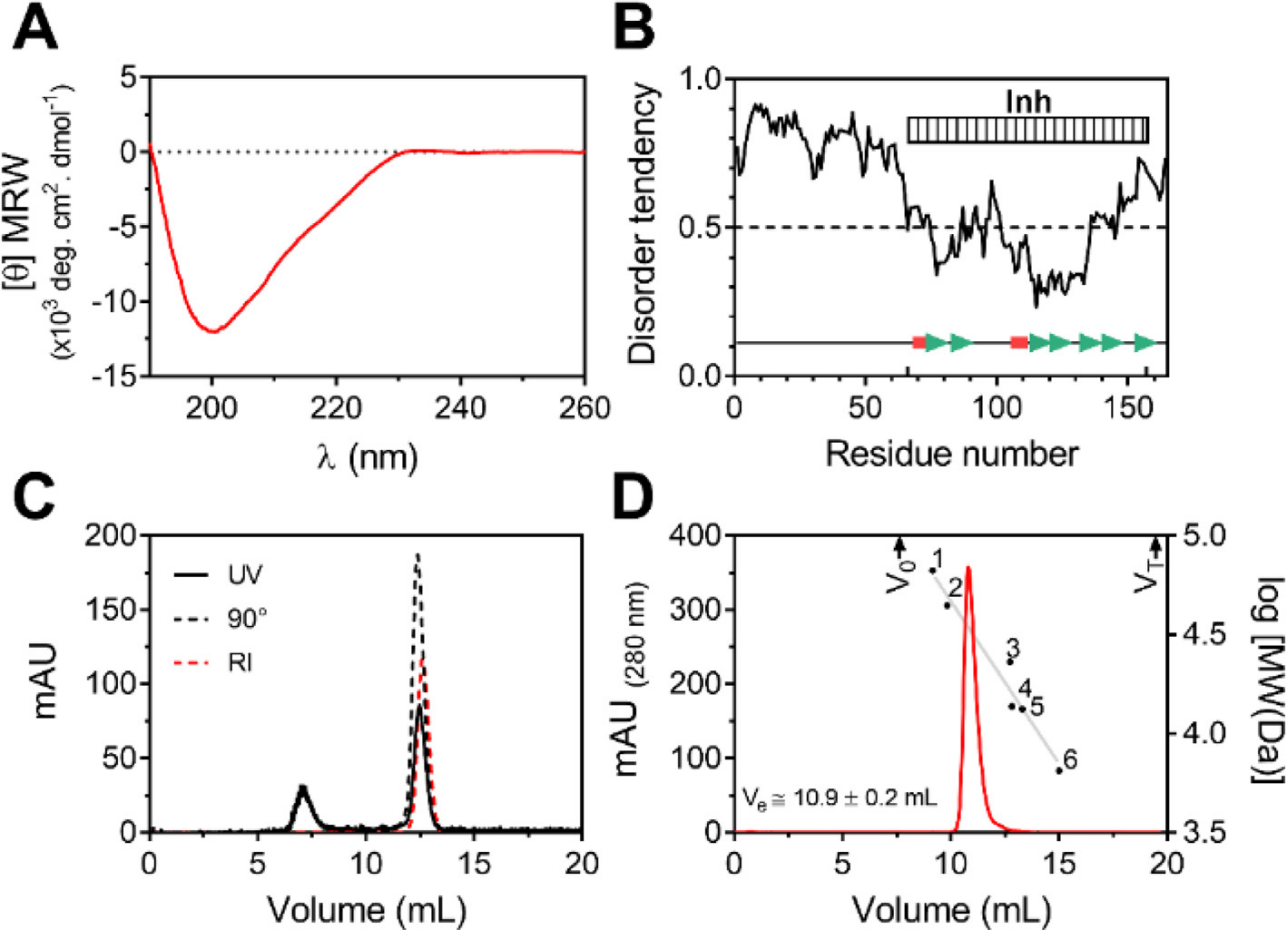
Full-length U-Omp19 is predicted to be composed of an extended disordered N-terminal and a globular C-terminal domain rich in β structure. (A) U-Omp19 Far-UV CD spectrum. (B) *In silico*-structural predictions. Intrinsically disordered regions (IDR) were predicted using the IUPred3 server (43). Results are shown as a score between 0 and 1 for each residue corresponding to the probability of the given residue being part of a disordered region. The secondary structure elements showed at the bottom were predicted using PSIPRED server (44). α-Helix or β-sheet secondary structures are highlighted as pink boxes or green arrows, respectively. The box at the top of the figure represents the region matching with the Pfam Inh domain (residues 66–157). (C) SEC-SLS analysis. The plot shows the chromatogram of U-Omp19. The solid line represents the UV signal, the black dashed line the LS signal, and the red dashed line the RI signal. (D) Size-exclusion chromatography profile. Elution volumes of the globular proteins used as standards are represented as: 1-BSA (66 kDa), 2-Ovalbumin (44 kDa), 3- Papain (23 kDa), 4-Ribonuclease A (13.7 kDa), 5-Staphostanin A (13.3 kDa) and 6-Aprotinin (6.5 kDa). Arrows at the top indicate the position of the void (V_0_) and the total (V_T_) volume. SLS and SEC experiments were carried out using different analytical Superdex 75 10/300 GL columns, where each run was performed in triplicate for all the samples analyzed and results are presented as mean ± SEM. (For interpretation of the references to color in this figure legend, the reader is referred to the web version of this article.)

To get insights into U-Omp19 secondary structure the Far-UV circular dichroism (CD) spectrum was obtained. The spectrum showed a significant negative signal in the 210–230 nm region compatible with high β-structure content. The presence of disordered regions was suggested by a deep minimum at 200 nm characteristic of natively unfolded or intrinsically disordered proteins (IDP) [41] (Fig. 1A). Deconvolution of the CD spectrum suggested that U-Omp19 is composed of 40.0 % of regular secondary structure (35.6 ± 1.2 % β-sheet and 4.4 ± 0.6 % α-helix), and of 60.0 ± 0.8 % of other structures.

U-Omp19 sequence analysis using IUPRED3 [42] and PSIPRED [43] servers predicted high propensity to disorder in the N-terminal region (residues 1–65) and predominantly β-sheet folding in C-terminal region (residues 66–159) that matched with the previously predicted Inh domain [7] (Fig. 1B).

To determine the molecular weight (MW) and oligomeric state in solution of U-Omp19 we conducted size-exclusion chromatography (SEC) coupled with static laser light scattering (SLS), refractive index (RI), and ultraviolet (UV) detection. U-Omp19 eluted as a single and symmetric peak with a MW of 16.6 ± 0.3 kDa, in close agreement with the value calculated from its sequence (MW_THEO_ = 16.8 kDa) (Fig. 1C). However, the elution volume of U-Omp19 was smaller than the expected for a globular protein with comparable MW (Fig. 1D). The ratio between the apparent MW determined by SEC (MW_SEC_) and the MW_THEO_ was significantly higher than 1 (2.08 ± 0.07, p < 0.005), indicating that the hydrodynamic behavior of U-Omp19 did not resemble that of a compact globular structure. Together, these results indicate that U-Omp19 is a monomer with an extended conformation in solution, compatible with the presence of a natively unfolded N-terminal region.

**Fig. 2:**
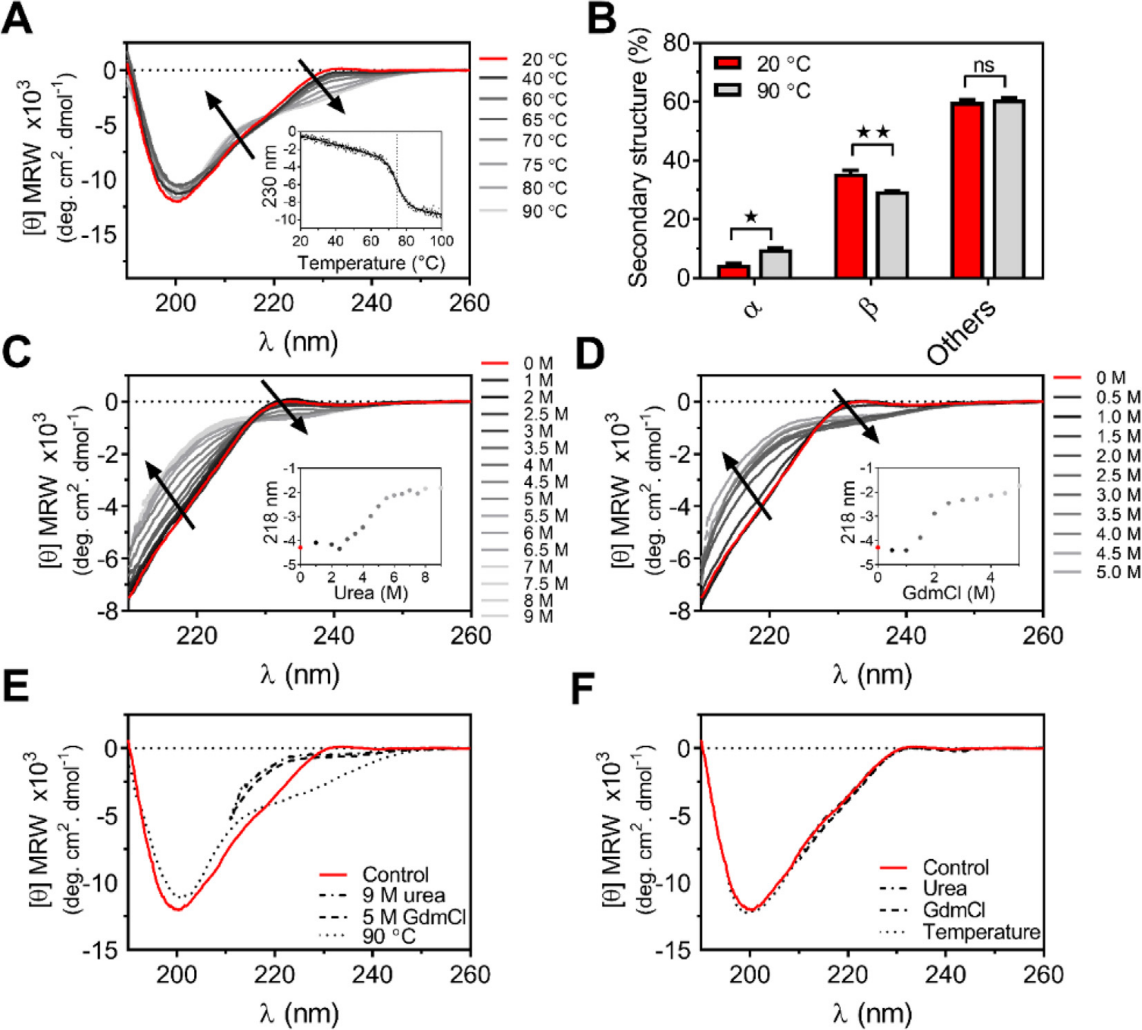
Effect of temperature and chaotropic agents on the secondary structure of U-Omp19. (A) Effect of increasing temperature on the secondary structure of U-Omp19. Far-UV CD spectra at different temperatures. The arrows represent the direction of the conformational change upon heating. Inset: Secondary structure changes were analyzed by monitoring the loss of CD signal at 230 nm while increasing temperatures from 20 to 100 °C. A vertical dashed line indicates the midpoint temperature of the thermal unfolding curve. (B) Comparison between the amount of secondary structure elements at 20 °C (red) and 90 °C (grey) as predicted by deconvolution of Far-UV CD spectra. Each bar represents the mean percentage (±SEM) of each secondary structure (α, β, and others). Data were analyzed by two-way ANOVA followed by Bonferroni’s multiple comparisons test, ns P > 0.05; ^★^P < 0.05; ^★★^P < 0.01 *vs* the 20 °C condition. (C-D) Chemical denaturation unfolding followed by Far-UV CD. Spectra of U-Omp19 titrated with 10.0 M urea (C) or 6.0 M GdmCl (D) at 20 °C. Black arrows represent the direction of changes in the spectra while increasing the concentration of the denaturing agent. Insets: Changes in ellipticity monitored at 218 nm during chemical denaturation. (E) Comparison of secondary sturcture changes on U-Omp19 subjected to high temperature or chemical denaturation. Superposition of Far-UV spectra of (i) the native protein at 20 °C (control), (ii) in presence of urea (9 M) or (iii) GdmCl (5 M) or (iv) after heated at 90 °C. (F) Reversibility of the structural transitions. Superposition of Far-UV CD spectra of the native protein (control), and the refolded proteins after (i) the heated protein was returned to room temperature or (ii) the protein in 9 M urea or 5 M GdmCl was diluted to the lowest concentration of the chemical agent. (For interpretation of the references to color in this figure legend, the reader is referred to the web version of this article.)

Far-UV CD spectra acquired at different temperatures confirmed that, as previously described (7), U-Omp19 retains high secondary structure content, even at 90 °C. Analysis of the secondary structure content at 20 or 90 °C suggested a moderate temperature-induced gain in α-structure and a minor loss of β-structure (Fig. 2A-B). As the temperature increased, changes in spectra occurred mostly in the 225–235 nm region. An isodichroic point was evidenced at 220 nm, with no significant loss of signal at 218–222 nm (Fig. 2A), suggesting that a local conformational transition occurred upon heating. Changes in the 225–235 nm region with heating have been attributed to aromatic and/or disulfide contributions and/or changes in polyproline type II structure [44], [45]. Analysis of the ellipticity at 230 nm as a function of temperature showed that the transition occurred in a cooperative manner with a midpoint centered at 74.6 ± 0.2 °C (Fig. 2A inset). Together these results indicate that while no global unfolding occurs with heating there is a local and reversible conformational transition that may involve differences in aromatic or cysteine residue environments.

Chemical denaturation by urea and guanidinium chloride (GdmCl) was also assessed. U-Omp19 Far-UV CD spectra were obtained at different concentrations of urea (Fig. 2C) or GdmCl (Fig. 2D) at 20 °C. In the presence of a high concentration of denaturants, U-Omp19 underwent a marked loss of secondary structure suggested by the loss of signal at 220 nm. The spectra recorded in the presence of 6 M urea or 4 M GdmCl were compatible with those of an unfolded protein (Fig. 2C-E). When plotting the ellipticity at 218 nm *vs* the denaturant concentration, it became clear that the major overall changes occurred between 3 and 5 M urea and 1–2 M GdmCl and were consistent with a cooperative global unfolding transition (Fig. 2C-D insets). The set of spectra obtained also presented isodichroic points compatible with a two-state behavior, however, these were found at higher wavelengths (230 nm) than in thermal denaturation (Fig. 2C-D). These results indicate that the structural transitions and the final states obtained by thermal and chemical denaturation were different, with only chemical denaturation causing global unfolding (Fig. 2E). In all cases, the transitions were reversible as the spectra acquired after return to room temperature or dilution of the denaturant agent were superimposable to the initial spectra (Fig. 2F).

### 3.2 NMR spectroscopy confirmed that U-Omp19 holds a disordered N-terminus and a C-terminal domain rich in β structure.

**Fig. 3:**
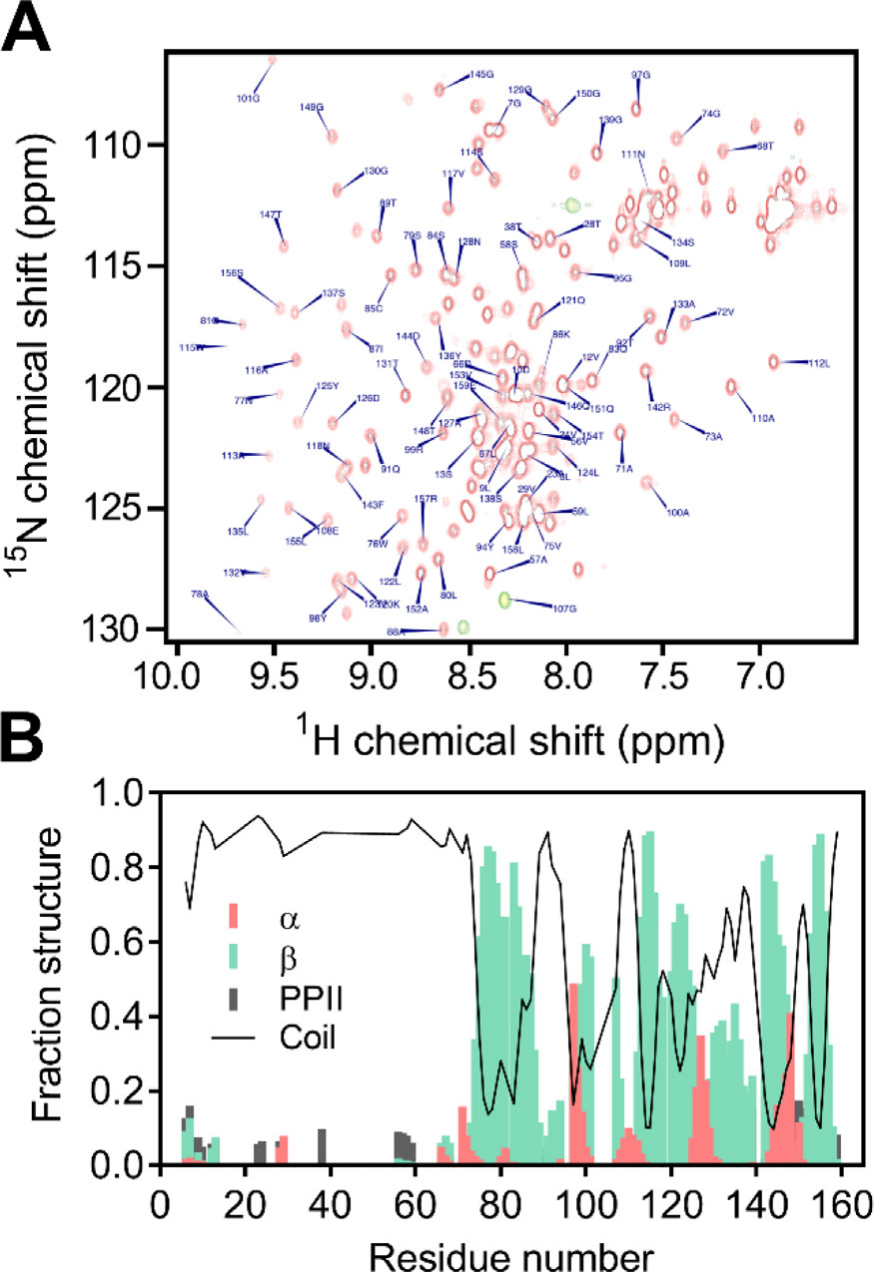
NMR characterization of U-Omp19. (A) ^1^H-^15^N-HSQC spectrum of U-Omp19 (red). Backbone resonance assignments for the full-length 165-residue ^13^C, ^15^N-uniformly labeled U-Omp19 were obtained using a standard set of triple resonance experiments. (B) Probability distributions of secondary structure element populations (α-helix, β-strand, random coil, and polyproline II), based on the information provided by backbone chemical shifts using the delta2d software. (For interpretation of the references to color in this figure legend, the reader is referred to the web version of this article.)

To obtain further information of the conformation of U-Omp19 in solution, its behavior by nuclear magnetic resonance (NMR) was studied. Fig. 3A shows the ^15^N-^1^H HSQC spectrum of ^13^C, ^15^N-U-Omp19 with resonance assignment. The mixture of well-dispersed resonances with several resonances clustered in the central region indicates the presence of both folded and unfolded regions, respectively. The unambiguous assignment of HN-N resonances was possible for 95 signals out of the expected 147 resonances. Analysis of the backbone secondary chemical shifts along the U-Omp19 sequence confirmed that the first 65 residues are disordered, while the rest of the protein adopts mostly β-sheet folding (Fig. 3B).

### 3.3 The crystal structure of the C-terminal domain of U-Omp19 shows a tightly packed β-barrel fold, which is conserved among members of the I38 family

In agreement with the XtalPred server [46] predictions that classified U-Omp19 within the protein class with lowest crystallizability, all attempts to crystallize it were unsuccessful. NMR analysis allowed to pinpoint flexible residues providing valuable information to improve the crystallizability. However, attempts to crystallize the C-terminal region of U-Omp19 [U-Omp19_(60-159)_] also failed. Of note, almost all crystallization conditions either for U-Omp19 or U-Omp19_(60-159)_ resulted in clear drops without evidence of protein precipitation, despite working with concentrations over 200 mg/ml, suggesting an extremely high solubility.

**Fig. 4:**
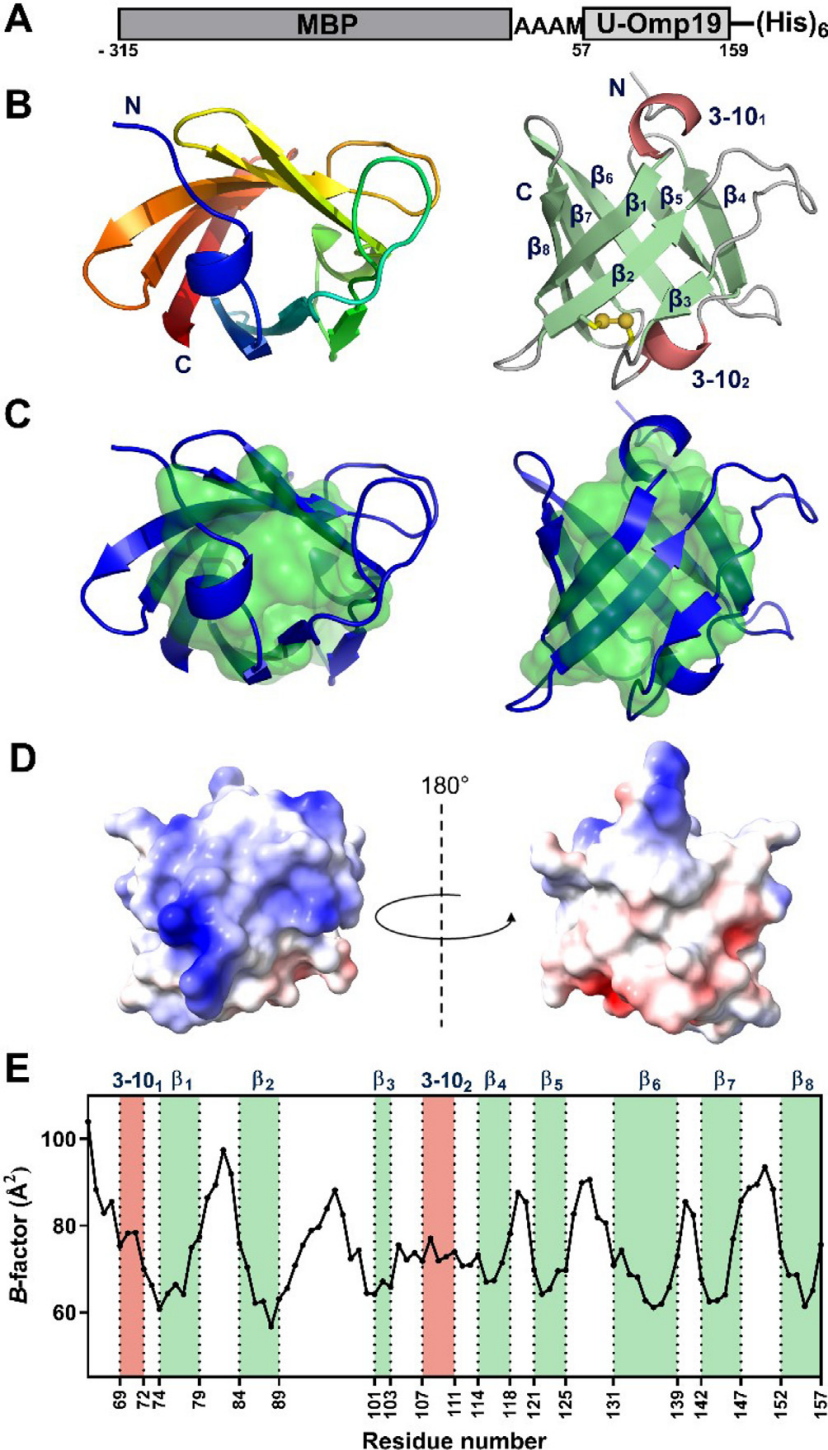
Crystal structure of MBP-U-Omp19_(57-159)_. (A) Schematic representation of the protein construct generated for crystallographic purposes. The C-terminal region of U-Omp19 (residues 57–159) with the histidine tag (residues 160–165) was fused to an engineered MBP. The amino acid numbering was assigned so that the residues of the U-Omp19 fraction coincide with those of the full-length protein. (B) Ribbon diagram of the tertiary structure of the C-terminal domain of U-Omp19 obtained by crystallography. The images show orthogonal views of the β-barrel core of U-Omp19. In the left panel the structure is represented color-coded from the N-terminus (blue) to the C-terminus (red), in the right panel the two 3–10-helices are showed in pink, β-strands in green and coils in grey. The β-strands are labeled from β_1_ to β_8_ according to the U-Omp19 sequence and the helices from 3-10_1_ to 3–10_2_. The disulfide bond Cys85-Cys105 is depicted in yellow. (C) Representation of the inner hydrophobic core (green) comprising the following residues: leucine [67, 80, 109, 112, 122, 124, 135], valine [72, 117, 132, 153], alanine [78, 100, 133], tryptophan [76, 115], cysteine [85 and 105], isoleucine [87], and phenylalanine [143]. Both pictures correspond to orthogonal views of the β-barrel core of U-Omp19 in the same orientation as panel B. (D) Representation of the electrostatic potential surface of the C-terminal domain of U-Omp19. The left panel has the same orientation as panels B and C right, and the right panel is rotated 180°as indicated. Positive potential is highlighted in blue and negative in red. (E) Crystallographic *B*-factor values specified per residue. Regions with either 3_10_-helical or β-sheet structure are highlighted in orange and green, respectively. (For interpretation of the references to color in this figure legend, the reader is referred to the web version of this article.)

To increase the likelihood of crystallization, we fused the predicted folded domain of U-Omp19 (residues 57–159) to an engineered maltose binding protein (MBP) [19] to obtain the MBP-U-Omp19_(57-159)_ construct (Fig. 4A). MBP-U-Omp19_(57-159)_ crystallized as described in Methods and its structure was solved and refined by molecular replacement method at 2.55 Å resolution with favorable geometry (Table 2). The asymmetric unit contains a single polypeptide chain. No electron density was observed for the N-terminal methionine of MBP (residue −315), the residue ranges −143 to −141 (MBP) and 62 to 64 (U-Omp19), and three histidine residues from the affinity tag. An exploration of the probable assemblies suggested by PDBePISA revealed that the fusion protein could be a dimer or a tetramer in solution with buried areas of 3730 and 12080 Å^2^, respectively. The putative dimer may be formed by a twofold symmetry axis that runs parallel to the c direction in the crystal packing, and which shows extensive contact between the Omp19 and MBP moieties from the neighboring chains (Fig. S1A). In this arrangement, the neighboring Omp19 moieties present an interface area of 520 Å^2^ involving the loop that connects strands β6 and β7 and residues from both their N- and C-termini (see Fig. 4B for the secondary structure nomenclature). The putative tetramer can be regarded as a dimer of dimers in which the Omp19 pairs interact mostly through strands β6 and β7 (Fig. S1B). It is important to stress that the MBP-Omp19_(57-159)_ fusion protein is a chimeric construct and, as such, its quaternary structure in solution and in the crystal may not necessarily reflect the quaternary arrangement of U-Omp19, which proved to be a monomer in solution, as shown before by SLS-SEC.

U-Omp19_(67-157)_ (∼size 25 × 30 × 30 Å^3^) is formed by eight antiparallel β-strands arranged in the shape of a compact β-barrel with two 3–10-helices capping both openings. The two cysteines form an intramolecular disulfide bridge that connects strand β2 to the loop between strand β3 and helix 3–10_2_ (Fig. 4B). The internal cavity of the β-barrel is tightly packed mostly by hydrophobic residues (Fig. 4C). No ordered solvent molecule, ligand nor any charged residue was found inside. Moreover, no internal cavity could be detected using a 1.4-Å radius probe. One side of the beta barrel presents a marked positive charge, whereas the opposite region shows a mixed tendency with a negative overall charge (Fig. 4D). The refined B-factor values showed that the connecting loops of the β-barrel exhibit higher flexibility than the β-strands, while both 3–10-helices present intermediate B-factor values (Fig. 4E).

**Fig. 5:**
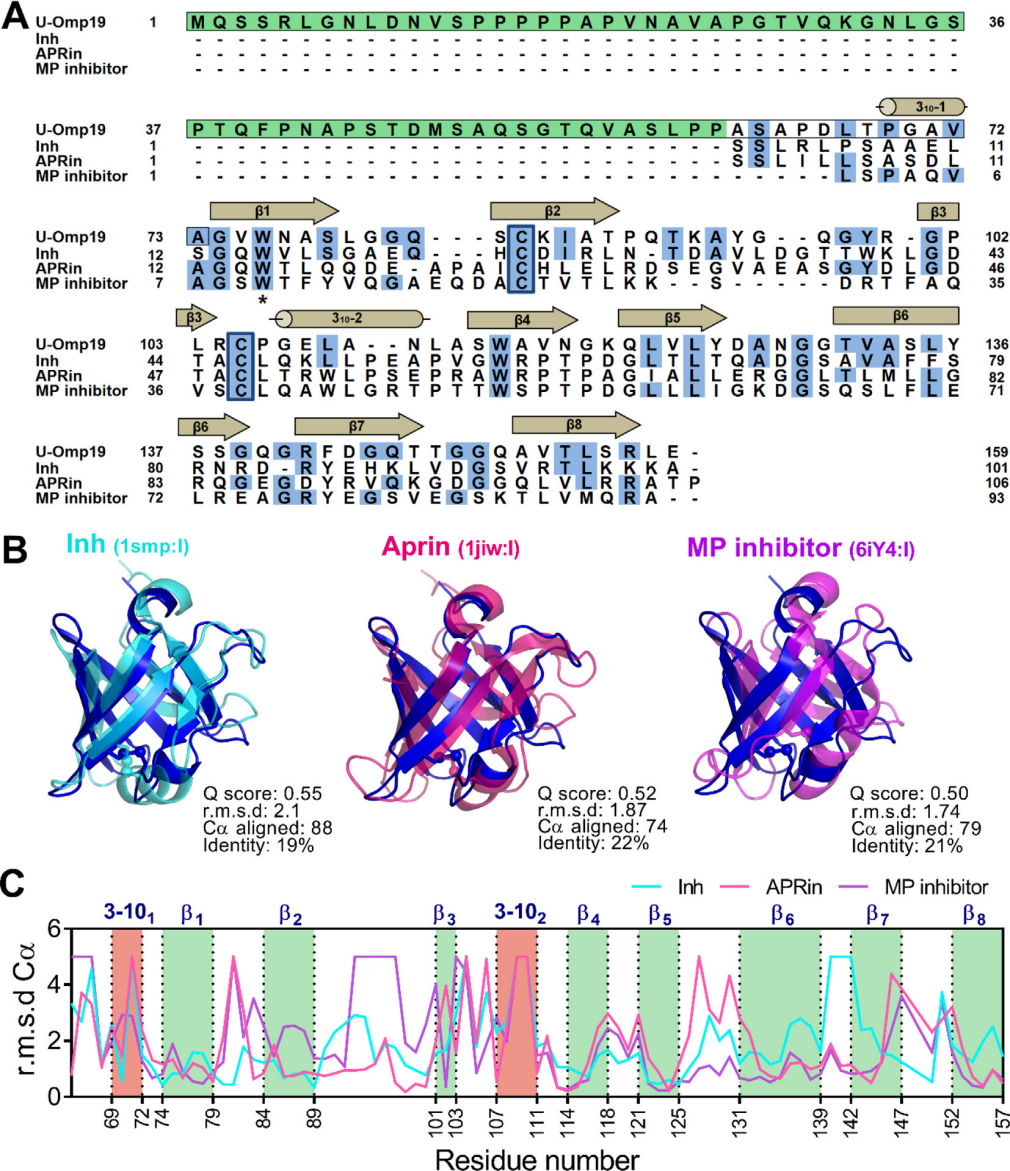
Sequence and structural alignment of U-Omp19 and its three closest structural matches.(A) Multiple sequence alignment of U-Omp19 and the three closest hits according to the PDBeFold server: (i) the inhibitor Inh from *E. chrysanthemi,* (ii) the alkaline protease inhibitor APRin of *P*. *aeruginosa* and (iii) the marine protease MP inhibitor from *Flavobacterium* sp. YS-80–122. Identical amino acids are highlighted in blue. Highly conserved cysteine residues among all sequences are depicted in blue boxes. Location of the secondary structure elements at the top of the alignment refers to the U-Omp19 crystal structure obtained in this work. The N-terminal domain (NTD) of U-Omp19 corresponds to the boxed sequence (residues 1 to 73). The asterisk shows the Trp15 of APRin that is conserved in all structures and has been related to the CD signal at 230 nm. The sequence highlighted in green corresponds to the variant: U-Omp19_(1–60)_ protein (see below). (B) Superposition between U-Omp19 (blue) and members of Inh family in a similar orientation to Fig. 4B (right). (C) Pairwise backbone heavy atom r.m.s.d (Å) between the U-Omp19 β-barrel and the Inh (1smp:I), APRin (1jiw:I) or MP inhibitor (6iy4:I) structures. (For interpretation of the references to color in this figure legend, the reader is referred to the web version of this article.)

A search for similar structures in the Protein Data Bank confirmed a high similarity between U-Omp19 and members of the I38 family. The three top hits are (i) Inh from *Erwinia chrysanthemi* [47], (ii) the alkaline protease inhibitor APRin of *Pseudomonas aeruginosa* [48] and (iii) the marine protease MP inhibitor from *Flavobacterium* sp. YS-80–122. Even though the C-terminal region of U-Omp19 shares low sequence identity with these three proteins (∼20 %) (Fig. 5A-B), their tridimensional structures are highly conserved (Fig. 5B). The disulfide bridge is highly conserved among all related structures, and the most variable regions correspond in general to the loops and the helix 3–10_2_ (Fig. 5 B-C). Altogether, these results indicate that the C-terminal domain of U-Omp19 has a tightly packed β-barrel folding conserved between members of the Inh family [7].

### 3.4 The C-terminal domain of U-Omp19 shows a restricted flexibility in solution along its sequence.

To get deeper information about the β-barrel domain structure and dynamics in solution, we pursued an NMR analysis of the U-Omp19_(60-159)_ construct. Ninety backbone amide signals out of 92 observed in the ^1^H-^15^N-HQSC spectrum of U-Omp19_(60-159)_ could be assigned. The backbone chemical shifts confirmed that the truncated version is well folded with 8 β-strands and a 3–10 helix between β3 and β4, in general agreement with the crystallographic results.

No significant shifts in the signals corresponding to the β-barrel in U-Omp19_(60-159)_ compared to those in U-Omp19 were found (Fig. S2), suggesting that no interactions occur between the N-terminal and C-terminal regions.

**Fig. 6:**
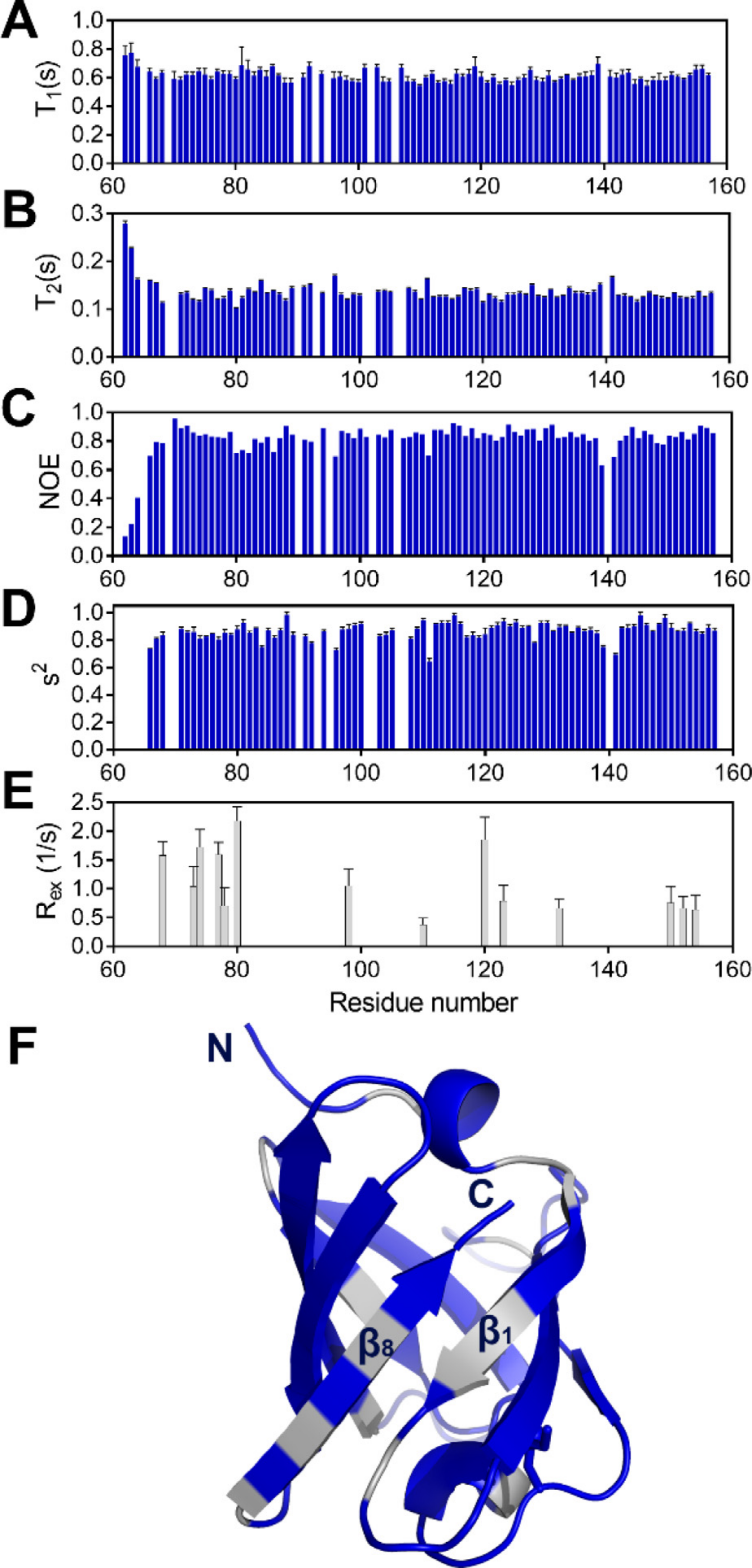
Dynamics of U-Omp19 C-terminal region derived from NMR analyses.Backbone ^15^N relaxation times (A) T1, (B) T2 and (C) heteronuclear ^1^H/^15^N NOE along the U-Omp19 C-terminal sequence. Backbone dynamics calculation by model free analysis was performed using T1, T2 and NOE. (D) Estimated order parameters (s^2^) and (E) Exchange contributions (R_ex_) per residue along the U-Omp19 C-terminal sequence. (F) Mapping of residues with exchange contributions (grey) on the crystal structure of U-Omp19 (blue). (For interpretation of the references to color in this figure legend, the reader is referred to the web version of this article.)

Backbone dynamics of U-Omp19_(60-159)_ by ^15^N relaxation measurements were then assessed (Fig. 6). Overall, the ^15^N-^1^H NOEs showed that all secondary structural elements and even the loops are mostly rigid on ps/ns timescales, while a modest flexibility was observed within Ala62-Ala64. These results are consistent with uniform backbone dynamics with restricted mobility and a high conformational order. Model free analysis of the T1, T2 and ^15^N-^1^H NOE relaxation data also showed overall high order parameters along the sequence but revealed a subset of residues with exchange contributions to T2. These residues cluster mostly in the first and final β-strands (Fig. 6F), suggesting some strain in the sewing point of the β-barrel.

### 3.5 The intramolecular disulfide bridge plays a role in U-Omp19 stabilization.

**Fig. 7:**
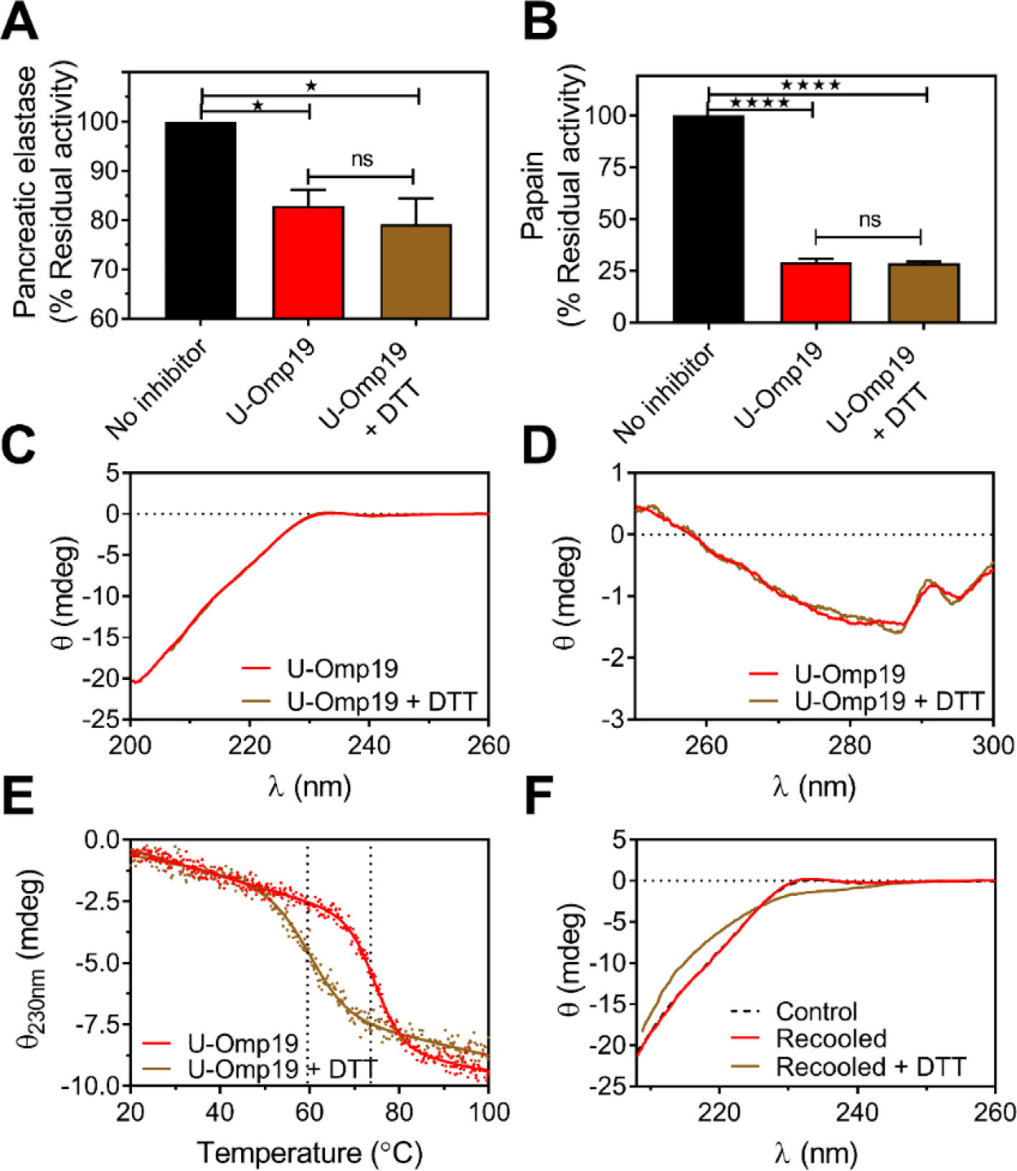
Role of the disulfide bridge in U-Omp19 activity, structure, and stability.Protease inhibitor activity of U-Omp19 in the presence or absence of reducing agent. Pancreatic elastase (A) or papain (B) were incubated for 1 h with U-Omp19 in the presence or absence of 1 mM DTT; or buffer (No inhibitor). The residual protease activity was determined after addition of specific protease fluorogenic substrates and is expressed as the percentage of protease activity remaining when compared to the “No inhibitor” condition (100 % of activity). Data were analyzed by one-way ANOVA followed by Bonferroni’s test, ns P > 0.05 *vs* U-Omp19 (without DTT) or ^★^P < 0.05; ^★★★★^P < 0.0001 *vs* “No inhibitor” condition. Far-UV CD spectrum recorded at 10 µM protein concentration (C) and Near-UV CD spectrum recorded at 100 µM protein concentration (D) of U-Omp19 in the presence (brown) or absence (red) of DTT. (E) Thermal denaturation profiles assessed by CD spectroscopy at 230 nm of the native (red) and DTT-treated (brown) U-Omp19 and the curves obtained were fit to a two-state model. (F) Reversibility of the thermal denaturation process of the native or reduced U-Omp19. Far-UV CD spectra assessed after the heated protein was returned to room temperature in the presence (brown) or absence (red) of DTT. The black line shows the Far-UV CD spectrum of U-Omp19 before heating as control. (For interpretation of the references to color in this figure legend, the reader is referred to the web version of this article.)

The inhibition of pancreatic elastase (Fig. 7A) and papain (Fig. 7B) by U-Omp19 was similar either in the presence or absence of reducing agent (DTT). Also, Far- and near-UV CD spectra of U-Omp19 were almost identical either in the presence or absence of DTT (Fig. 7C-D). Nevertheless, the midpoint (Tm) of the thermal transition curve followed by Far-UV CD (74.6 ± 0.2 °C) shifted significantly towards a lower temperature when DTT was added (59.5 ± 0.4 °C) (unpaired *t*-test, P = 0.0004) (Fig. 7E). Furthermore, the thermal induced structural transition in U-Omp19 was fully reversible in the absence but not in the presence of DTT, evidenced by a subtle change in the Far-UV CD spectrum obtained after cooling the reduced protein (Fig. 7F). Altogether these results indicate that while disruption of the disulfide bond neither alters the protease inhibitor activity nor the secondary/tertiary structure of folded U-Omp19, the disulfide bridge plays a role in stabilizing the local secondary and possibly tertiary structure during heating. A similar structural transition was described for APRin and was attributed to its Trp15 residue [49], which is conserved in U-Omp19 (Trp76) (Fig. 5A). The stabilizing role of the disulfide bond in this transition is compatible with the short distance between it and the Trp76 in the β-barrel structure (Fig. S3).

### 3.6 U-Omp19 requires its N-terminal region for its protease inhibitor activity

**Fig. 8:**
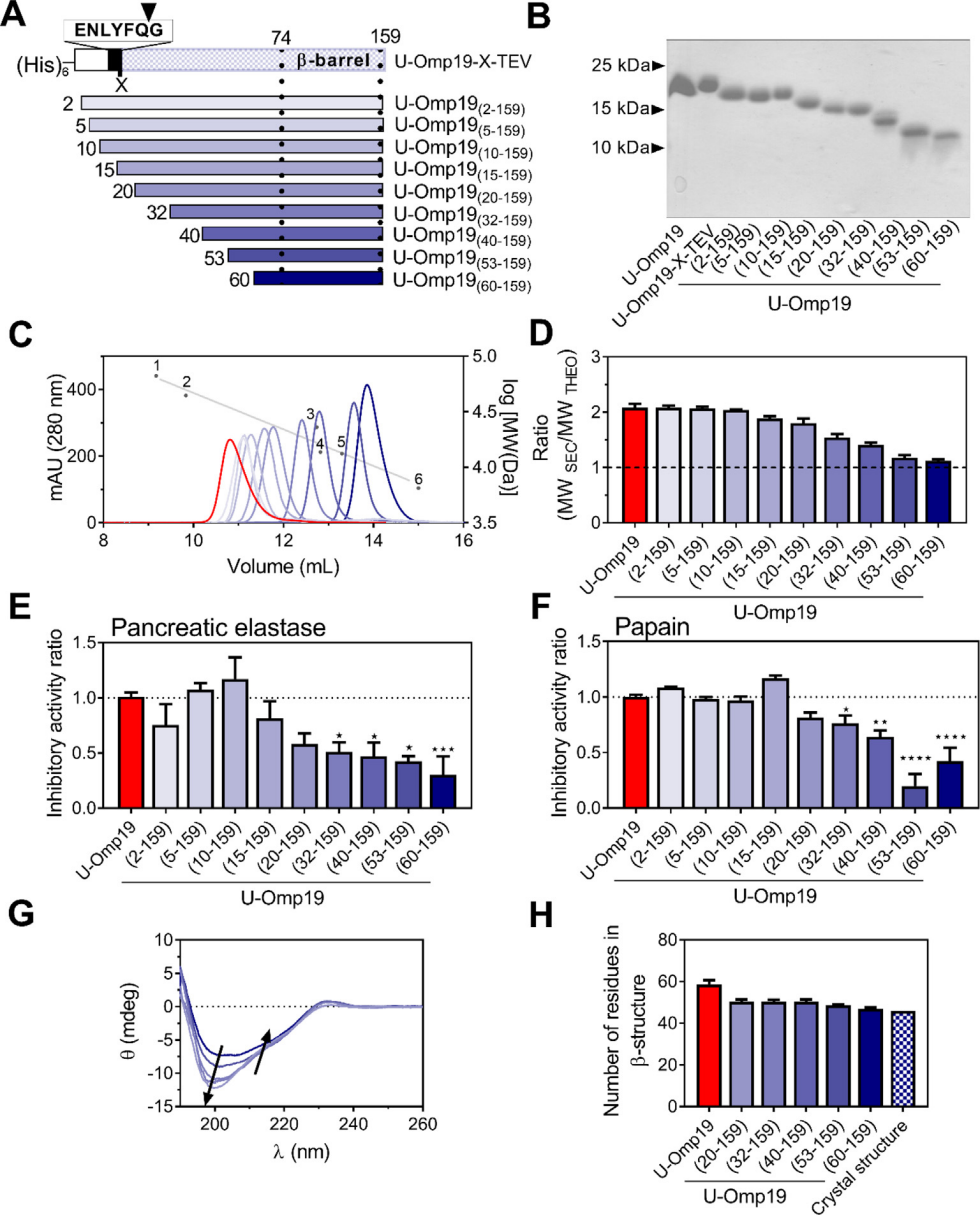
The intrinsically disordered N-terminal region of U-Omp19 is required for the protease inhibitor activity of U-Omp19. (A) Schematic representation of the U-Omp19-X-TEV constructs designed to obtain N- terminal truncated variants. The boxed sequence shows the TEV recognition heptapeptide inserted in the recombinant proteins at the “X” residue (the residue numbering is referred to the 165-residue U-Omp19 protein). The arrow at the top shows the cleavage site by TEV protease. The fragments of interest of the full-length protein are depicted as the colored segment after the inserted sequence. Bellow the U-Omp19-X-TEV, the nine variants obtained in this work are represented. The regions between the dashed lines correspond to the β-barrel domain. (B) 18 % SDS–PAGE Coomassie-stained gel of truncated U-Omp19 variants under reduced conditions. (C) SEC profiles of U-Omp19_(X-159)_ versions compared to U-Omp19 (red). Individual chromatograms are superimposed for presentation. Elution volumes of the globular proteins used as standards are represented as in Fig. 1D. Each sample was analyzed in triplicate. (D) Ratio between MW_SEC_ and MW_THEO_ for each truncated protein. The bar graph shows the mean ± SEM, the dashed line highlights the expected ratio for a globular protein (MW_SEC_/MW_THEO_ ∼1). Protease inhibitor activity of pancreatic elastase (E) or papain (F) was determined as in Fig. 7. Bar graphs show the inhibitor activity ratio between inhibitor activity of each tested protein and the inhibitor activity of full-length U-Omp19 (Inhibitor activity of U-Omp19_(X-159)_ / Inhibitor activity of Full-length U-Omp19). A ratio = 1 indicates that the variant retains the full inhibitory activity, while a ratio < 1 indicates loss of inhibitory activity. Data were analyzed by one-way ANOVA followed by Bonferroni’s test, ^★^P < 0.05; ^★★^P < 0.01; ^★★★^P < 0.001; ^★★★★^P < 0.0001 *vs* U-Omp19 inhibition. (G) Far-UV CD spectra of U-Omp19_(X-159)_ proteins. Arrows indicate the direction of the change in the secondary structure in the Far-UV CD spectra of the C-terminal domains as the length of the protein increases and approaches the full-length U-Omp19 spectrum. (H) Comparison of the number of residues with β-secondary structure obtained from the deconvolution of the CD spectra of panel G whit that calculated from the crystal structure. (For interpretation of the references to color in this figure legend, the reader is referred to the web version of this article.)

In protease inhibitors of the I38 family, the β-barrel and the ∼10-residue N-terminal region linked to it are essential for protease inhibitor activity [50], [51]. Since the N-terminal region of U-Omp19 is significantly longer than those of other I38 family members, and to identify the minimal fragment that retains the protease inhibitor activity, we designed constructs with N-terminal deletions. Nine different constructs [U-Omp19_(X-159)_] were generated by inserting a Tobacco Etch Virus (TEV) protease cleavage sequence at specific points (residue X = 2, 5, 10, 15, 20, 32, 40, 53, 60) of U-Omp19 (Fig. 8A, Table 1). The shortest construct, U-Omp19_(60-159)_, encompassed the β-barrel (residues 74–159) plus 14 residues of the N-terminus covering the sequence shared with the I38 family members (Fig. 5A). SDS-PAGE analysis showed that the expression, cleavage, and purification processes resulted in truncated proteins with the expected MWs (Fig. 8B, Table 1).

Each U-Omp19_(X-159)_ eluted as a single and symmetric peak when subjected to SEC (Fig. 8C). The MW_SEC_/MW_THEO_ ratios were higher than 1 and decreased as larger regions were deleted. The ratio was close to 1 for U-Omp19_(60-159)_ indicating that it behaves like a compact globular protein (Fig. 8D).

Truncated proteins lacking up to their first 15 residues retained the full capacity of U-Omp19 to inhibit pancreatic elastase or papain (Fig. 8E-F). On the contrary, truncated proteins lacking more than the first 20 residues gradually lost their inhibitor activity against both proteases. This reduction was statistically significant when 32 or more residues were removed (Fig. 8E-F), indicating that the N-terminal region, encompassing residues 20–60, is required for the protease inhibitor activity of U-Omp19.

Far-UV CD spectra of the truncated proteins confirmed that they were properly folded (Fig. 8G). The number of residues predicted to have β-structure according to the BeStSel server was conserved among proteins and agreed with those calculated from the crystal structure (Fig. 8H). In addition, NMR data indicated that the β-barrel structure of U-Omp19_(60-159)_ and U-Omp19 are similar (Fig. S2). Consequently, there is no evidence of loss of folded structure on the β-barrel upon deletion of the N-terminal region. Besides, deletion of the first 60 residues of U-Omp19 had neither an effect on the Tm of the thermal transition at 230 nm nor in the midpoint of the urea denaturation and refolding curves extracted from Far-UV CD spectra (Fig. S4), suggesting that deletion of up to 60 residues of the N-terminus does not significantly destabilize the β-barrel fold. Altogether these results indicate that the N-terminal region is required for the inhibitor activity of U-Omp19, and that the loss of inhibitor activity of the truncated proteins is not likely due to changes in the folded structure of the β-barrel.

### 3.7 The disordered N-terminal region of U-Omp19 is responsible for its protease inhibitor activity

To test the contribution of the N-terminal region to the protease inhibitor activity of U-Omp19, we obtained the fragment between Met1 and Pro60, [U-Omp19_(1–60)_].

**Fig. 9:**
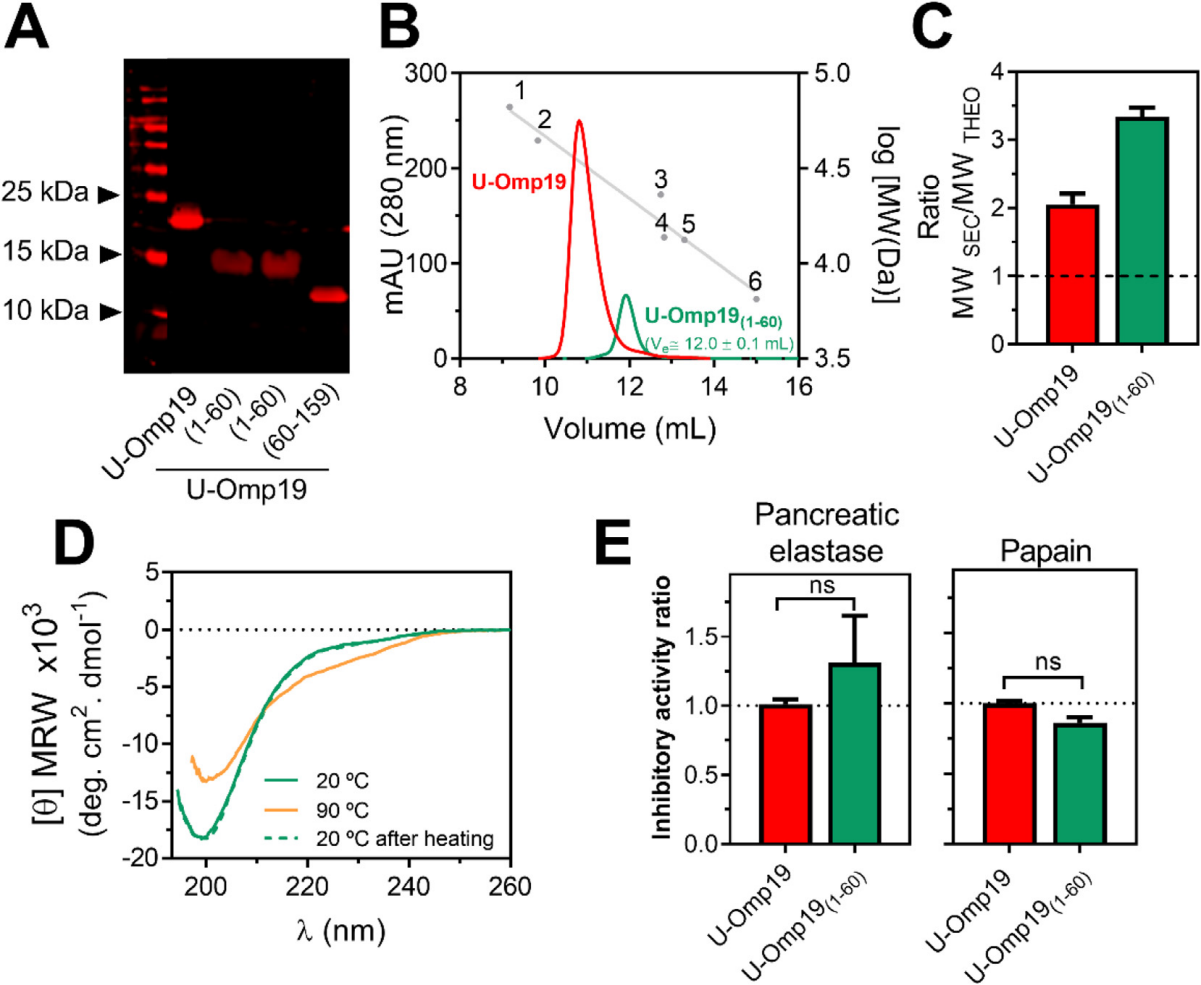
The intrinsically disordered N-terminal region is the main determinant of U-Omp19 protease inhibitor activity. (A) Western blot analysis of U-Omp19_(1–60)_ (MW_THEO_ = 6835.5) using a polyclonal rabbit anti-U-Omp19 serum. Lane 1: MW standards. Lane 2: U-Omp19, Lane 3: U-Omp19_(1–60)_, Lane 4: U-Omp19_(1–60)_ with no heating in Laemmli buffer before running into the SDS-PAGE gel. Lane 5: U-Omp19_(60-159)_. (B) SEC profile of U-Omp19_(1–60)_ compared to full-length U-Omp19_._ Elution volumes of the globular proteins used as standards are represented as in Fig. 1D. The figure is a representative chromatogram of three independent measurements. (C) Hydrodynamic properties of U-Omp19_(1–60)_. The bar graph shows the MW_SEC_/MW_THEO_ parameter for each depicted protein. The dashed line indicates the MW_SEC_/MW_THEO_ for ideal globular proteins (∼1). (D) Far-UV CD spectra of U-Omp19_(1–60)_ at 20 °C (green solid line), at 90 °C (orange solid line) or of the heated protein at 90 °C after re-cooling to 20 °C (green dashed line). (E) Protease inhibitor activity of U-Omp19_(1–60)_ against pancreatic elastase (left) or papain (right) were performed as described in Fig. 8E-F. Bar graphs represent inhibitor activity ratio calculated as the ratio of inhibitor activity of the tested protein and inhibitor activity of full-length U-Omp19. A ratio = 1 indicates retention of full inhibitory activity, while a ratio < 1 indicates loss of inhibitory activity. Data were analyzed unpaired student *t*-test; ns P > 0.05 *vs* U-Omp19 activity. (For interpretation of the references to color in this figure legend, the reader is referred to the web version of this article.)

U-Omp19_(1–60)_ was not detectable by Coomassie Brilliant Blue staining probably due to its amino acid composition, which is depleted of “order-promoting” residues and enriched in most “disorder-promoting” residues [52], [53], that lead to a weak dye binding [54]. Also, U-Omp19_(1–60)_ showed anomalous electrophoretic mobility as revealed by Western blot (Fig. 9A). Although U-Omp19_(1–60)_ presented the electrophoretic migration of a globular protein of ∼15 kDa (Fig. 9A), mass spectrometry studies indicated a MW of 6836.4 Da (data not shown), compatible with its theoretical MW_THEO_ of 6835.6 Da. SEC analysis showed a single and symmetric peak that eluted as a globular protein of higher MW (Fig. 9B) with a high MW_SEC_/MW_THEO_ ratio of 3.36 ± 0.05 (Fig. 9C) that is consistent with the IDP character of U-Omp19_(1–60)_.

The Far-UV CD spectrum of U-Omp19_(1–60)_ was compatible with that of an IDP with a minimum near 200 nm and low ellipticity at 222 nm (Fig. 9D). However, the small negative signal at 222 nm suggests some residual secondary structure content. Analysis of the ellipticity at 222 *vs* 200 nm allowed to classify U-Omp19_(1–60)_ within the random coil subclass of IDPs [55]. The Far-UV CD spectrum acquired at 90 °C showed a decrease in signal at 200 nm and the characteristic of IDPs increase in ellipticity in the 220–240 nm region [45]. The structural transition induced by heat was modest and reversible (Fig. 9D). The arithmetic sum of the individual Far-UV CD spectra of the complementary pairs U-Omp19_(60-159)_ and U-Omp19_(1–60)_ was identical to the spectrum of full-length U-Omp19 (Fig. S5) suggesting that both fragments are structurally independent, in agreement with the previous NMR and Far-UV CD results.

Surprisingly, U-Omp19_(1–60)_ retained the full inhibitory activity of full-length U-Omp19 against pancreatic elastase, papain (Fig. 9E) and pepsin (Fig. S6), indicating that this region is not only required, but is also sufficient for the protease inhibitor activity of U-Omp19.

### 3.8 The N-terminal region of U-Omp19 retains the adjuvant activity for oral co-delivered Ags.

**Fig. 10:**
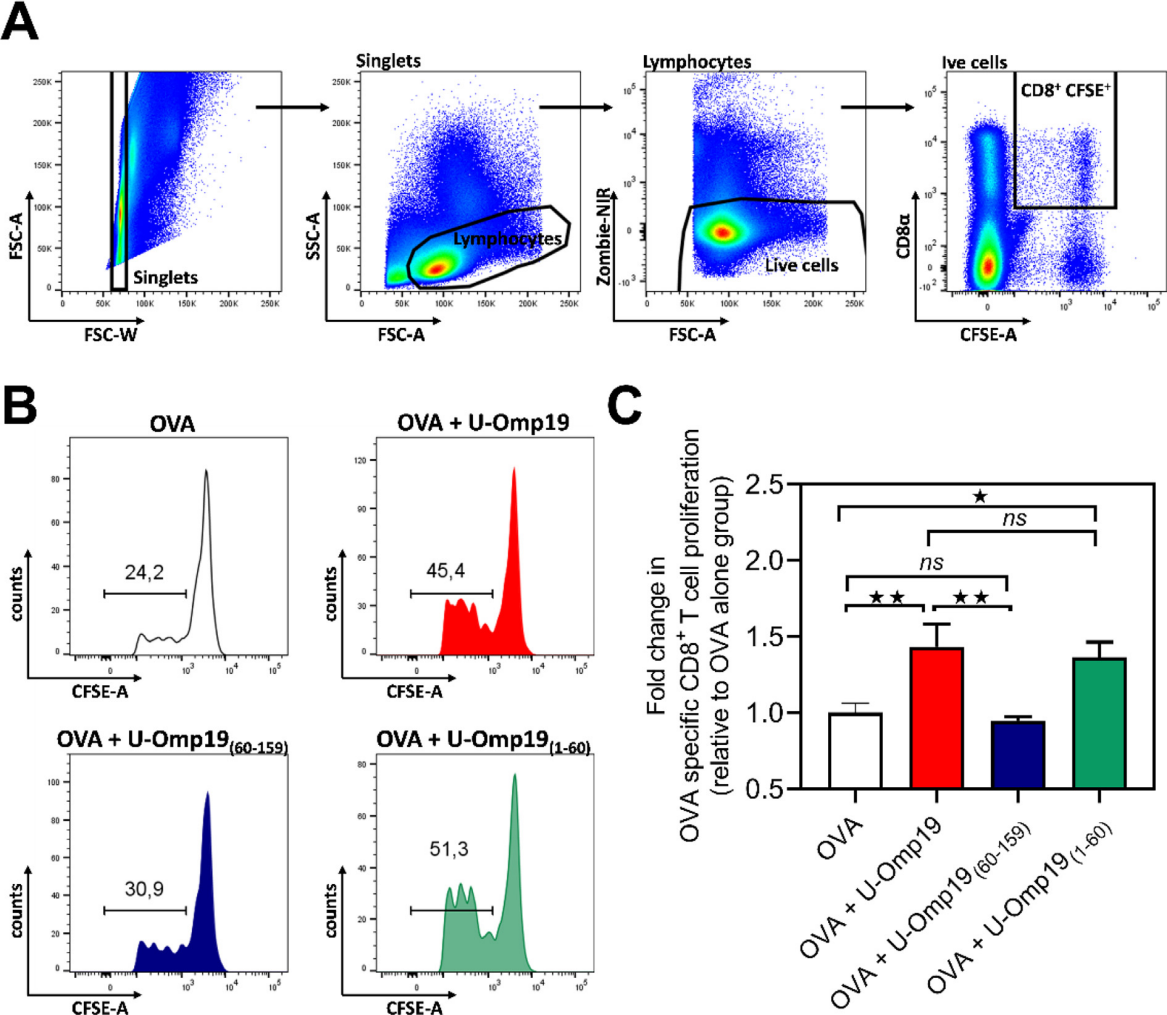
The U-Omp19 NTD retains the full adjuvant activity of U-Omp19 *in vivo.* C57BL/6 mice were adoptively transferred with CFSE labeled lymphocytes of OT-I transgenic mice. On the next day groups of mice were immunized by gavage with a single dose of (i) OVA, (ii) OVA + U-Omp19, (iii) OVA + U-Omp19_(60-159)_, (iv) OVA + U-Omp19_(1–60)_, or (v) saline. Three days after immunization the proliferation of OVA specific CD8^+^ T cells was analyzed in the spleens by flow cytometry. (A) Gating strategy used to select the CFSE^+^ CD8^+^ T cells. (B) Representative histograms showing CFSE dilution in CFSE^+^ CD8^+^ T cells in each immunization groups. The marker indicates the proportion of proliferating cells of the selected population CFSE^+^ CD8^+^ T cells (C) Fold change in OVA specific CD8^+^ T cells proliferation relative to the group immunized with OVA alone. Data represent pooled results of three independent experiments with n = 2 to 4/group for each trial. Statistical analyses were performed using one-way ANOVA followed by Bonferroni posttest, ns P > 0.05; ^★^P < 0.05; ^★★^P < 0.01; ^★★★^P < 0.001; ^★★★★^P < 0.0001 *vs* OVA or OVA + U-Omp19 condition.

To evaluate the role of both complementary regions in the ability of U-Omp19 to increase the immune response to oral co-delivered Ags, we performed an *in vivo* T cell proliferation assay. CFSE-labeled OVA-specific CD8^+^ T cells were adoptively transferred into congenic C57BL/6 mice. One day later mice were immunized orally with OVA alone or OVA plus equimolar quantities of i) U-Omp19, ii) U-Omp19_(60-159)_ or iii) U-Omp19_(1–60)_. After three days the proliferation of OVA-specific CD8^+^ T cells was evaluated. The adjuvant activity of U-Omp19 was evidenced by the significant increase in the proliferation of OVA-specific CD8^+^ T cells in OVA + U-Omp19 compared to OVA alone-immunized mice. U-Omp19_(1–60)_ fully conserved this adjuvanticity since the increase in proliferation was similar when OVA was co-delivered either with U-Omp19 or U-Omp19_(1–60)_. In contrast, U-Omp19_(60-159)_ was not able to increase the assessed proliferation (Fig. 10). These results indicate that the first 60 residues of U-Omp19 are necessary and sufficient for the ability of U-Omp19 to increase the proliferation of Ag specific CD8^+^ T cells when administered orally with an Ag.

## 4 Discussion

Development of mucosal vaccine formulations and mucosal adjuvants has been a challenge for immunologists (1). Our group has been working on the development of a novel oral vaccine adjuvant called U-Omp19 which is a bacterial protease inhibitor with immunostimulatory properties [5], [6], [7], [8], [9], [10], [11], [12], [13]. However, the molecular properties of U-Omp19 and the contribution of each part of the molecule to its adjuvant and protease inhibitor activities remained unknown.

Structural biology is of utmost importance in biopharmaceutical products development, like adjuvants. Elucidating their structure–activity relationship may lead to understand their mechanism of action, and thus, help to move novel adjuvants from the bench to the clinic. In this work we focused on getting insights into structure–activity relationship of U-Omp19.

Crystallization of U-Omp19_(57-159)_ fused to MBP confirmed that the region spanning residues Pro69 to Arg157 adopts a typical eight-stranded β-barrel topology. NMR dynamics studies of U-Omp19_(60-159)_ confirmed that the β-barrel domain has a fairly rigid structure in solution like that revealed by X-ray structural analysis. The similarity between both structures suggest that the interactions in the crystal lattice are not inducing disorder-to-order transitions that result in static structures that significantly differ from those in solution, as has been shown for other proteins [25].

The U-Omp19 β-barrel has a striking structural similarity to the Inh domain. Proteins with this domain belong to family I38 [15], a group of bacterial protease inhibitors that are secreted into the periplasm where their presumed function is to protect proteins from the action of secreted metalloproteases from the serralysin branch of the metzincin superfamily [50], [51]. Major members of this family are (i) Inh [47] (ii) APRin [51] and (iii) the *Serratia marcescens* protease inhibitor SMPI [56]. These inhibitors fold into an eight-stranded β-barrel preceded by a ∼ 10-residue N-terminal region. Residues 1–5 prevent the access of the substrate by occluding the active site of the protease. The β-barrel prevents the penetration of the N-terminal region further than 5 residues into the protease substrate binding cleft and itself interacts with the protease by one face formed by the strands β3, β4 and β5 [47], [51].

As members of I38 family, U-Omp19 is capable of inhibiting proteases [7], [9]. However, unlike them that are soluble in the periplasm, Omp19 is anchored to the outer membrane of *Brucella* spp [57]. Currently, no metalloprotease of *Brucella* has been found to be inhibited by Omp19. Also, U-Omp19 inhibits serralysin with less potency than APRin (Fig. S7). Instead, U-Omp19 inhibits gastrointestinal- and intracellular-proteases [7], [9] and has been shown to play a role in protecting the bacterium from host proteases during the establishment of the infection [17].

The sequence identity between the U-Omp19 β-barrel and those of the related I38-family members is close to the nominal threshold for a reliable alignment. Nevertheless, the sequence identity among the three known members of I38 family is also low [51]. However, unlike the rest of the inhibitors, whose N-terminal regions of ∼10 residues are conserved, the entire N-terminus of U-Omp19 is significantly longer and spans 73 residues. Even with this low sequence identity, the tertiary structure of the β-barrel including the position of the disulfide bond is highly conserved.

Protease inhibitors of the I38 family are closely related to lipocalins, fatty acid-binding proteins, avidins and triabins. Together, these families constitute the calycin superfamily that shares a common β-barrel core structure. These proteins are characterized by their ability to bind specific cell-surface receptors, to form complexes with soluble macromolecules and to hold small hydrophobic molecules in the internal ligand-binding site of the β-barrel [58]. However, the U-Omp19 hydrophobic cavity is too crowded to contain any molecule. In addition to members of the I38 family, other proteins within the calycin superfamily are protease inhibitors like the tear lipocalin [59] or triabin, which inhibits thrombin by blocking its fibrinogen-binding exosite by hydrophobic interactions that involve three β-sheets of the β-barrel [60]. Staphostatin A and B are also protease inhibitors that adopt an eight-stranded β-barrel structure like Inh [15], [50]. These inhibitors compete with the substrate for binding to the active site of staphopains. The interaction involves a loop of the β-barrel that binds the active site cleft of the protease and a hydrogen bond between two antiparallel beta β-sheets that stabilizes the bound conformation and prevents the hydrolysis of the loop in the active site cleft [61]. Together, this evidence reveals a broad diversity of protease inhibitory mechanisms involving β-barrel domains.

Thus, we decided to test the role of the β-barrel domain in protease inhibition of U-Omp19 using a set of truncated proteins with N-terminal deletions. The shortest construct, U-Omp19_(60-159)_, included the β-barrel plus 14 residues of the N-terminal disordered region that matches approximately the length of the N-terminal region involved in the inhibitory activity of the I38-family members [51], [62]. This short region also contains a conserved serine residue (Ser63) that in APRin and Inh was reported to interact with the catalytic glutamate residue of the metalloproteases [51] and the 3–10_1_ helix (residues 69 to 72) that is also well conserved in the Inh family and has been involved in the interaction between APRin and APR [49]. However, unexpectedly, U-Omp19_(60-159)_ significantly lost the ability to inhibit pancreatic elastase, papain and pepsin. The analysis of the inhibitor activity of the complete set of constructs indicated that residues 20–60 are required for most protease inhibitor activity.

The isolated N-terminal fragment U-Omp19_(1–60)_ presented anomalous electrophoretic mobility in SDS-PAGE. Although anomalous migration in SDS-PAGE can be attributed to the high content of acidic residues and thus low binding of SDS [63], other parameters such as expansion in solution [64] and a high proline content [65] can contribute to anomalous migration. This is probably the case for U-Omp19_(1–60)_, since its acidic content does not differ significantly from that of globular proteins and its expansion inferred from SEC experiments is compatible with a random coil conformation [55]. This intrinsically disordered and highly expanded U-Omp19_(1–60)_ N-terminal fragment has demonstrated to be necessary and sufficient to encode the full inhibitory activity of U-Omp19 towards pancreatic elastase (serine-protease), papain (cysteine-protease) and pepsin (aspartic-protease). Even though the enzymes examined are not the metalloproteases specifically inhibited by the I38 family, the mechanism of inhibition of U-Omp19 differs from these inhibitors. U-Omp19 is the first example within the I38 family in which the mechanism of protease inhibition does not involve the β-barrel domain and is fully associated to an intrinsically disordered region. Remarkably, U-Omp19_(1–60)_ not only encodes the protease inhibitor activity, but also retains the ability to stimulate CD8^+^ T cell responses to orally co-delivered Ags [7], [9]. Hence, since both activities reside in the intrinsically disordered region, it is likely that the adjuvant activity of U-Omp19 is linked to its ability to inhibit proteases. However, this is not a unique requisite for adjuvanticity, since not all protease inhibitors have the ability to increase immune responses [7], [9].

The different regions of U-Omp19 may also play roles other than inhibition of proteases in the adjuvanticity of U-Omp19, such as recruitment and activation of DCs, the interaction with an unknown membrane or intracellular receptor or with other cell types. In addition, the interaction of the adjuvant with the Ag could also play a role in immunogenicity and the different parts of the molecule may differentially interact with the Ag. Moreover, the use of this mouse model for the evaluation of adjuvant activity retention, although useful [66], does not cover all T and B cell adjuvant activities that have been previously demonstrated for U-Omp19. Therefore, further work is needed to elucidate the complete molecular mode of action of U-Omp19.

Currently, few IDPs have been reported as protease inhibitors or regulators of proteolysis [67]. Among them, calpastatin is an intrinsically unstructured calpain inhibitor that undergoes a folding transition upon binding to the cysteine protease [68]. Tryptogalinin is a tick serine protease inhibitor [69] that lacks one of the three highly conserved disulfide bridges of the Kunitz family [70]. This feature increases its intrinsic disorder and has been related to a broader spectrum and greater affinity against additional serine proteases [69].

Some IDPs can bind several different partners in a process called binding promiscuity [71]. Likewise, U-Omp19 inhibits different types of proteases with different mechanisms of action. While U-Omp19 inhibits serine-proteases in a mixed noncompetitive manner [7], it inhibits cysteine-proteases by a competitive mechanism [9]. The existence of multiple conformations for the disordered N-terminus of U-Omp19 may be related to its ability to inhibit different proteases [7], [9]. Otherwise, since short peptides have been shown to have protease inhibitor activities [72], [73], different peptides from the N-terminal domain of U-Omp19 may mediate the inhibition of the different proteases. However, the mechanism implied in the interaction with proteases is still unclear, and further studies on protein–protein interactions are required.

Overall, we report a structural, biochemical, and functional characterization of U-Omp19. Dynamic features of U-Omp19 in solution and the crystal structure of its C-terminal region are revealed. The structural organization of this adjuvant consists in a flexible intrinsically disordered N-terminus and a compact and globular β-barrel domain. We also show that the isolated intrinsically disordered N-terminal domain encodes the full protease inhibitor and adjuvant activities of U-Omp19, revealing a novel inhibitory mechanism compared to structurally related protease inhibitors.

## Author contributions

MLD: experimental design, methodology of most experiments, data interpretation and writing. CPC: methodology of in vivo adjuvanticity studies, LMC: experimental design of in vivo adjuvanticity studies. LB: methodology of in vivo adjuvanticity studies and protein purification; MLC: methodology of SLS and SEC experiments. LHO: crystallographic data collection. LBC: Experimental design, methodology and data interpretation of structural stability and conformational properties as assessed CD and SEC. RMR: experimental design, methodology, data interpretation, writing and reviewing of NMR experiments results. SK: experimental design, methodology, data interpretation, writing and reviewing of protein crystallization studies. JC: conceptualization, funding, and reviewing. KAP: conceptualization, funding, experimental design, supervision, data analysis, original draft preparation and writing.

## Declaration of Competing Interest

The authors declare the following financial interests/personal relationships which may be considered as potential competing interests: KAP, LMC and JC are inventors of a patent related to U-Omp19 “Adjuvant for vaccines, vaccines that comprise it and uses thereof” PCT/ES2010/070667. The owner of this patent is the National Research Council CONICET. The existence of the patent did not have any role in experimental design, data collection and analysis, decision to publish, or preparation of this manuscript. The remaining authors declare that the research was conducted in the absence of any commercial or financial relationships that could be construed as a potential conflict of interest.

## References

[1] Lavelle E.C., Ward R.W. (2021). Mucosal vaccines — fortifying the frontiers. Nat Rev Immunol.

[2] Pulendran B., Arunachalam P.S., O’Hagan D.T. (2021). Emerging concepts in the science of vaccine adjuvants. Nat Rev Drug Discovery.

[3] Del Giudice G., Rappuoli R., Didierlaurent A.M. (2018). Correlates of adjuvanticity: a review on adjuvants in licensed vaccines. Semin Immunol.

[4] McKee A.S., Marrack P. (2017). Old and new adjuvants. Curr Opin Immunol.

[5] Coria L.M., Martinez F.L., Bruno L.A., Pasquevich K.A., Cassataro J. (2020). U-Omp19 from Brucella abortus increases dmLT immunogenicity and improves protection against Escherichia coli heat-labile toxin (LT) oral challenge. Vaccine.

[6] Risso G.S. (2017). U-Omp19 from Brucella abortus Is a Useful Adjuvant for Vaccine Formulations against Salmonella Infection in Mice. Front Immunol.

[7] Ibanez A.E. (2015). A bacterial protease inhibitor protects antigens delivered in oral vaccines from digestion while triggering specific mucosal immune responses. J Control Release.

[8] Coria L.M. (2016). Brucella abortus Omp19 recombinant protein subcutaneously co-delivered with an antigen enhances antigen-specific T helper 1 memory responses and induces protection against parasite challenge. Vaccine.

[9] Coria L.M. (2016). A Brucella spp. protease inhibitor limits antigen lysosomal proteolysis, increases cross-presentation, and enhances CD8+ T cell responses. J Immunol.

[10] Caeiro L.D. (2020). The Trypanosoma cruzi TcTASV-C protein subfamily administrated with U-Omp19 promotes a protective response against a lethal challenge in mice. Vaccine.

[11] Coria L.M. (2019). Oral co-administration of a bacterial protease inhibitor in the vaccine formulation increases antigen delivery at the intestinal epithelial barrier. J Control Release.

[12] Coria L.M. (2022). A novel bacterial protease inhibitor adjuvant in RBD-based COVID-19 vaccine formulations containing alum increases neutralizing antibodies, specific germinal center B cells and confers protection against SARS-CoV-2 infection in mice. Front Immunol.

[13] Zhao J. (2021). A novel oral rabies vaccine enhances the immunogenicity through increasing dendritic cells activation and germinal center formation by expressing U-OMP19 in a mouse model. Emerg Microbes Infect.

[14] Coria LM, et al., A novel bacterial protease inhibitor adjuvant in RBD-based COVID-19 vaccine formulations increases neutralizing antibodies, specific germinal center B cells and confers protection against SARS-CoV-2 infection. bioRxiv 10.1101/2021.12.07.471590, 2021.2012.2007.471590 (2021). 10.3389/fimmu.2022.844837 PMC8919065 35296091

[15] Rawlings N.D. (2018). The MEROPS database of proteolytic enzymes, their substrates and inhibitors in 2017 and a comparison with peptidases in the PANTHER database. Nucleic Acids Res.

[16] Abbenante G., Fairlie D.P. (2005). Protease inhibitors in the clinic. Med Chem.

[17] Pasquevich K.A. (2019). Omp19 enables Brucella abortus to evade the antimicrobial activity from host's proteolytic defense system. Front Immunol.

[18] Darriba M.L., Cerutti M.L., Bruno L., Cassataro J., Pasquevich K.A. (2021). Stability studies of the vaccine adjuvant U-Omp19. J Pharm Sci.

[19] Moon A.F., Mueller G.A., Zhong X., Pedersen L.C. (2010). A synergistic approach to protein crystallization: combination of a fixed-arm carrier with surface entropy reduction. Protein Sci.

[20] Kapust R.B. (2001). Tobacco etch virus protease: mechanism of autolysis and rational design of stable mutants with wild-type catalytic proficiency. Protein Eng.

[21] Artimo P. (2012). ExPASy: SIB bioinformatics resource portal. Nucleic Acids Res.

[22] Micsonai A. (2018). BeStSel: a web server for accurate protein secondary structure prediction and fold recognition from the circular dichroism spectra. Nucleic Acids Res.

[23] Myers J.K., Pace C.N., Scholtz J.M. (1995). Denaturant m values and heat capacity changes: relation to changes in accessible surface areas of protein unfolding. Protein Sci.

[24] Estrada J., Bernado P., Blackledge M., Sancho J. (2009). Protsa. a web application for calculating sequence specific protein solvent accessibilities in the unfolded ensemble. BMC Bioinf.

[25] Uversky V.N., Dunker A.K. (2010). Understanding protein non-folding. Biochim Biophys Acta.

[26] Kabsch W. (2010). Xds. Acta Crystallogr D Biol Crystallogr.

[27] Evans P.R., Murshudov G.N. (2013). How good are my data and what is the resolution?. Acta Crystallogr Section D, Biol Crystallogr.

[28] Liebschner D. (2019). Macromolecular structure determination using X-rays, neutrons and electrons: recent developments in Phenix. Acta Crystallogr Section D, Struct Biol.

[29] Terwilliger T.C. (2008). Iterative model building, structure refinement and density modification with the PHENIX AutoBuild wizard. Acta Crystallogr D Biol Crystallogr.

[30] Emsley P., Lohkamp B., Scott W.G., Cowtan K. (2010). Features and development of Coot. Acta Crystallogr D Biol Crystallogr.

[31] Williams C.J. (2018). MolProbity: more and better reference data for improved all-atom structure validation. Protein Sci.

[32] Delaglio F. (1995). NMRPipe: a multidimensional spectral processing system based on UNIX pipes. J Biomol NMR.

[33] Vranken W.F. (2005). The CCPN data model for NMR spectroscopy: development of a software pipeline. Proteins.

[34] Markley J.L. (1998). Recommendations for the presentation of NMR structures of proteins and nucleic acids. IUPAC-IUBMB-IUPAB Inter-Union Task Group on the Standardization of Data Bases of Protein and Nucleic Acid Structures Determined by NMR Spectroscopy. J Biomol NMR.

[35] Lescop E., Schanda P., Brutscher B. (2007). A set of BEST triple-resonance experiments for time-optimized protein resonance assignment. J Magn Reson.

[36] Camilloni C., De Simone A., Vranken W.F., Vendruscolo M. (2012). Determination of secondary structure populations in disordered states of proteins using nuclear magnetic resonance chemical shifts. Biochemistry.

[37] Lipari G., Szabo A. (1982). Model-free approach to the interpretation of nuclear magnetic resonance relaxation in macromolecules. 1. Theory and range of validity. J Am Chem Soc.

[38] Clore G.M. (1990). Deviations from the simple two-parameter model-free approach to the interpretation of nitrogen-15 nuclear magnetic relaxation of proteins. J Am Chem Soc.

[39] Cole R., Loria J.P. (2003). FAST-Modelfree: a program for rapid automated analysis of solution NMR spin-relaxation data. J Biomol NMR.

[40] Pettersen E.F. (2021). UCSF ChimeraX: Structure visualization for researchers, educators, and developers. Protein Sci.

[41] Uversky V.N. (2002). What does it mean to be natively unfolded?. Eur J Biochem.

[42] Erdos G., Pajkos M., Dosztanyi Z. (2021). IUPred3: prediction of protein disorder enhanced with unambiguous experimental annotation and visualization of evolutionary conservation. Nucleic Acids Res.

[43] Buchan D.W.A., Jones D.T. (2019). The PSIPRED Protein Analysis Workbench: 20 years on. Nucleic Acids Res.

[44] Woody R.W. (1994). Contributions of tryptophan side chains to the far-ultraviolet circular dichroism of proteins. Eur Biophys J: EBJ.

[45] Chemes L.B., Alonso L.G., Noval M.G., de Prat-Gay G. (2012). Circular dichroism techniques for the analysis of intrinsically disordered proteins and domains. Methods Mol Biol.

[46] Slabinski L. (2007). XtalPred: a web server for prediction of protein crystallizability. Bioinformatics.

[47] Baumann U., Bauer M., Letoffe S., Delepelaire P., Wandersman C. (1995). Crystal structure of a complex between Serratia marcescens metallo-protease and an inhibitor from Erwinia chrysanthemi. J Mol Biol.

[48] Hege T., Feltzer R.E., Gray R.D., Baumann U. (2001). Crystal structure of a complex between Pseudomonas aeruginosa alkaline protease and its cognate inhibitor: inhibition by a zinc-NH2 coordinative bond. J Biol Chem.

[49] Gray R.D., Trent J.O. (2005). Contribution of a single-turn alpha-helix to the conformational stability and activity of the alkaline proteinase inhibitor of Pseudomonas aeruginosa. Biochemistry.

[50] Kantyka T., Rawlings N.D., Potempa J. (2010). Prokaryote-derived protein inhibitors of peptidases: a sketchy occurrence and mostly unknown function. Biochimie.

[51] Feltzer R.E., Gray R.D., Dean W.L., Pierce W.M. (2000). Alkaline proteinase inhibitor of Pseudomonas aeruginosa. Interaction of native and N-terminally truncated inhibitor proteins with Pseudomonas metalloproteinases. J Biol Chem.

[52] Williams RM, et al., The protein non-folding problem: amino acid determinants of intrinsic order and disorder. Pacific Symposium on Biocomputing. Pacific Symposium on Biocomputing 10.1142/9789814447362_0010, 89-100 (2001). 10.1142/9789814447362_0010 11262981

[53] Tompa P. (2002). Intrinsically unstructured proteins. Trends Biochem Sci.

[54] Sieber T. (2011). Intrinsic disorder in the common N-terminus of human adenovirus 5 E1B–55K and its related E1BN proteins indicated by studies on E1B–93R. Virology.

[55] Uversky V.N. (2002). Natively unfolded proteins: a point where biology waits for physics. Protein Sci.

[56] Kim K.S., Kim T.U., Kim I.J., Byun S.M., Shin Y.C. (1995). Characterization of a metalloprotease inhibitor protein (SmaPI) of Serratia marcescens. Appl Environ Microbiol.

[57] Tibor A., Decelle B., Letesson J.J. (1999). Outer membrane proteins Omp10, Omp16, and Omp19 of Brucella spp. are lipoproteins. Infect Immun.

[58] Flower D.R., North A.C., Sansom C.E. (2000). The lipocalin protein family: structural and sequence overview. BBA.

[59] Grzyb J., Latowski D., Strzalka K. (2006). Lipocalins - a family portrait. J Plant Physiol.

[60] Fuentes-Prior P. (1997). Structure of the thrombin complex with triabin, a lipocalin-like exosite-binding inhibitor derived from a triatomine bug. PNAS.

[61] Filipek R., Potempa J., Bochtler M. (2005). A comparison of staphostatin B with standard mechanism serine protease inhibitors. J Biol Chem.

[62] Bae K.H., Kim I.C., Kim K.S., Shin Y.C., Byun S.M. (1998). The Leu-3 residue of Serratia marcescens metalloprotease inhibitor is important in inhibitory activity and binding with Serratia marcescens metalloprotease. Arch Biochem Biophys.

[63] Weinreb P.H., Zhen W., Poon A.W., Conway K.A., Lansbury P.T. (1996). NACP, a protein implicated in Alzheimer's disease and learning, is natively unfolded. Biochemistry.

[64] Blocquel D., Habchi J., Gruet A., Blangy S., Longhi S. (2012). Compaction and binding properties of the intrinsically disordered C-terminal domain of Henipavirus nucleoprotein as unveiled by deletion studies. Mol BioSyst.

[65] Schramm A. (2019). An arsenal of methods for the experimental characterization of intrinsically disordered proteins – how to choose and combine them?. Arch Biochem Biophys.

[66] Lirussi D. (2018). Rapid <em>In Vivo</em> assessment of adjuvant's cytotoxic T lymphocytes generation capabilities for vaccine development. J Visualized Experiments.

[67] Dunker A.K., Brown C.J., Lawson J.D., Iakoucheva L.M., Obradovic Z. (2002). Intrinsic disorder and protein function. Biochemistry.

[68] Mucsi Z., Hudecz F., Hollosi M., Tompa P., Friedrich P. (2003). Binding-induced folding transitions in calpastatin subdomains A and C. Protein Sci.

[69] Valdes J.J. (2013). Tryptogalinin is a tick Kunitz serine protease inhibitor with a unique intrinsic disorder. PLoS ONE.

[70] Ranasinghe S., McManus D.P. (2013). Structure and function of invertebrate Kunitz serine protease inhibitors. Dev Comp Immunol.

[71] Kriwacki R.W., Hengst L., Tennant L., Reed S.I., Wright P.E. (1996). Structural studies of p21Waf1/Cip1/Sdi1 in the free and Cdk2-bound state: conformational disorder mediates binding diversity. PNAS.

[72] de Almeida Barros R. (2021). Small peptides inhibit gut trypsin-like proteases and impair Anticarsia gemmatalis (Lepidoptera: Noctuidae) survival and development. Pest Manag Sci.

[73] Xu P., Huang M. (2020). Small Peptides as Modulators of Serine Proteases. Curr Med Chem.

[74] Engh R.A., Huber R. (1991). Accurate bond and angle parameters for X-ray protein structure refinement. Acta Crystallogr Section A.

