# Supplementary material for: A disordered region retains the full protease inhibitor activity and the capacity to induce CD8+ T cells in vivo of the oral vaccine adjuvant U-Omp19
Source: Comput Struct Biotechnol J. 2022 Sep 6;20:5098–114. doi: 10.1016/j.csbj.2022.08.054 (PMC9486555; doi:10.1016/j.csbj.2022.08.054)
Supplement: Supplementary data 1 [file mmc1.docx]

1. **Supplementary material**

**Fig. S1.**


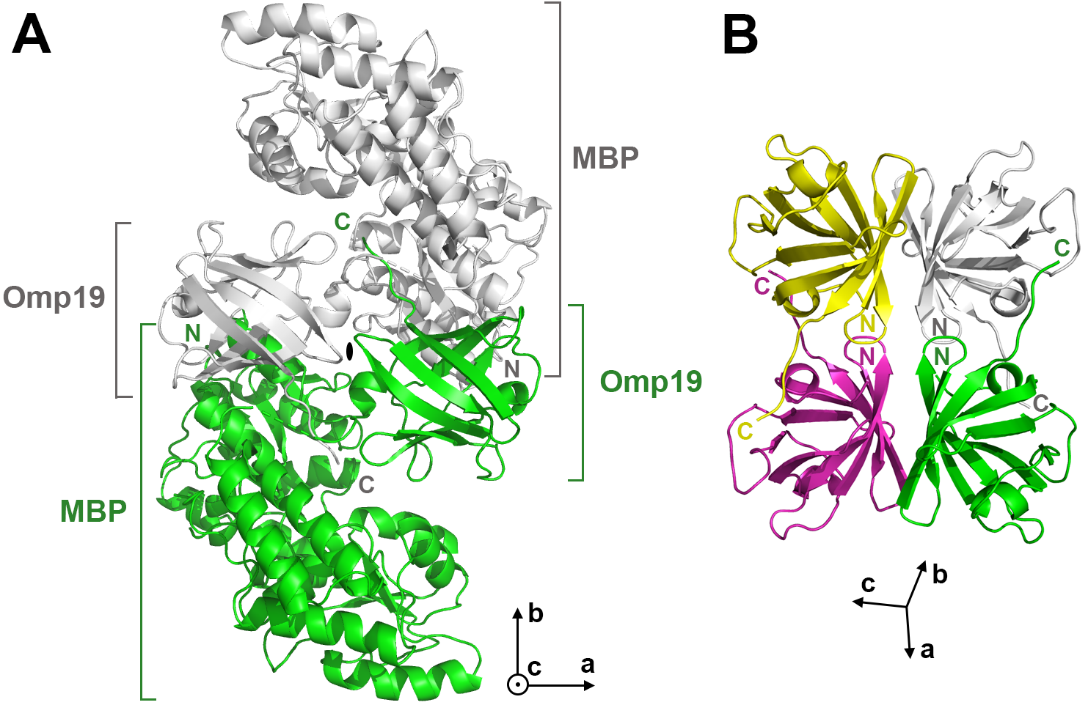


**Fig. S1.** **(A)** Putative dimeric arrangement of the fusion protein. The content of the asymmetric unit is depicted in green, and its symmetric partner is shown in gray. The Omp19 and MBP moieties are indicated for each chain. N- and C- termini are shown. The two-fold symmetry axis is depicted as a black oval at the center of the figure. The crystal axes are shown at the bottom. **(B)** Putative tetrameric arrangement of the fusion protein. Only the U-Omp19 moieties are shown for clarity. The U-Omp19 moieties in yellow and magenta correspond to the neighboring dimer.

**Fig. S2.**


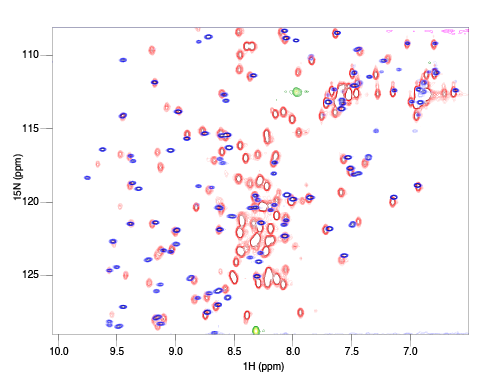


**Fig. S2.** ^1^H-^15^N HSQC NMR spectra of ^13^C, ^15^N- labeled U-Omp19 (red) or ^13^C, ^15^N- labeled U-Omp19_(60-159)_ (blue). Chemical shifts of the β-barrel domain show little variation in the absence of the N-terminal region of the protein.

**Fig. S3.**

**
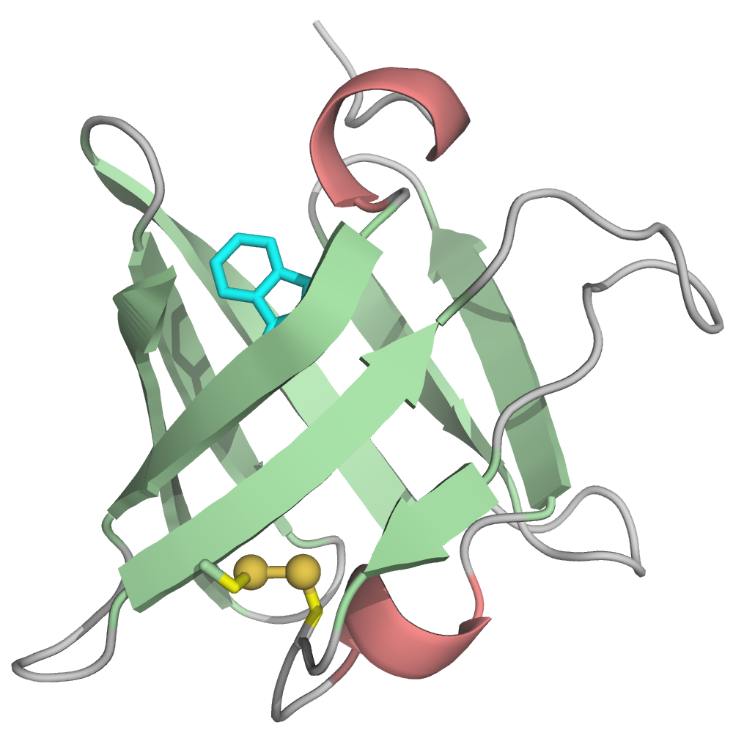
**

**Fig. S3. Location of Trp76 in the U-Omp19 crystal structure.** The figure highlights the side chain of Trp76, which is conserved in the other structures related to U-Omp19, and which corresponds to Trp15 in Aprin. This residue has been shown to contribute substantially to the signal at 230 nm in the Far-UV CD spectrum of Aprin and is consistent with the region of the spectrum subject to the greatest variation during the thermal transition of U-Omp19. Note the proximity of this residue to the disulfide bridge.

**Fig. 4.**


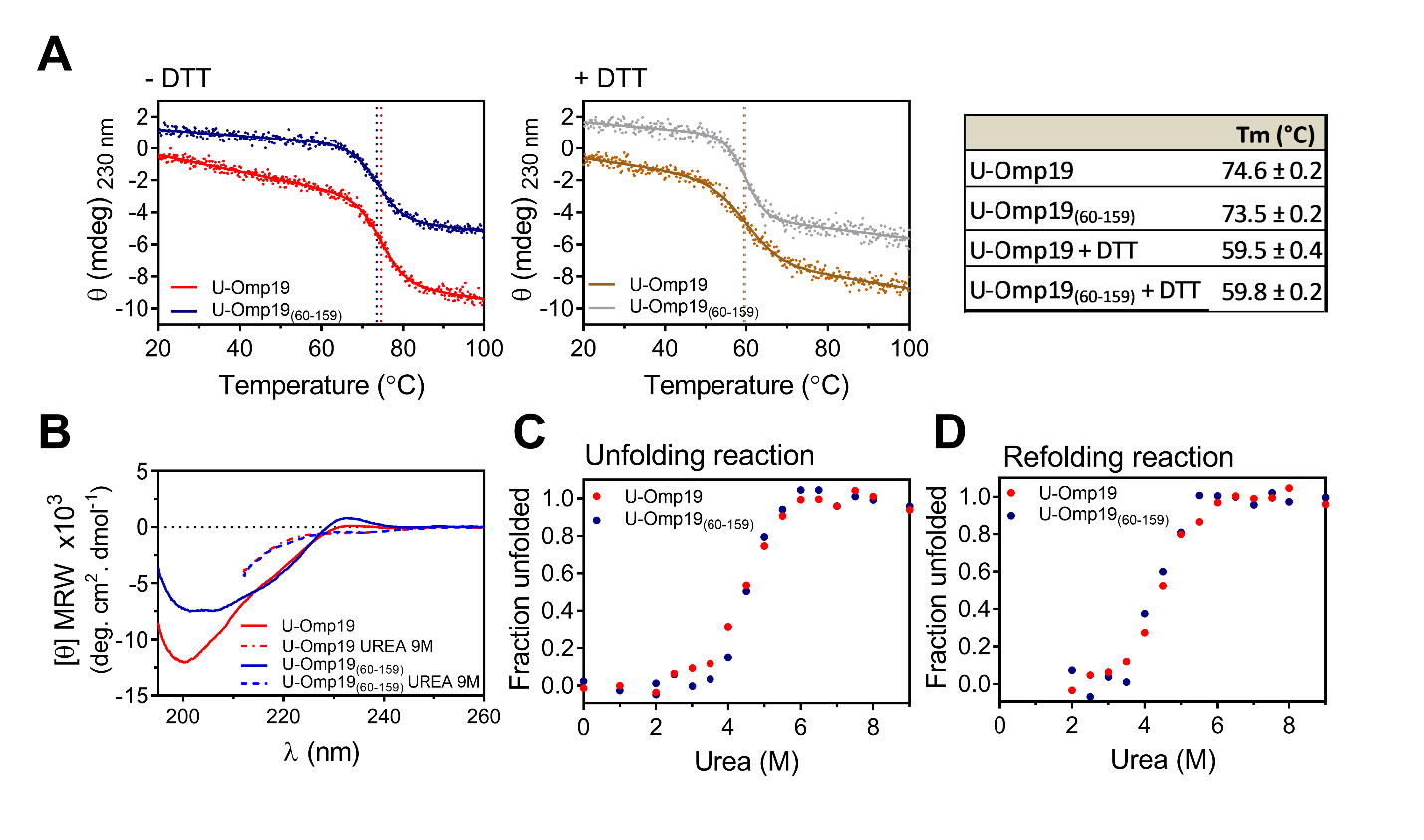


**Fig. S4. Comparison of the thermal and chemical denaturant-triggered structural transitions of U-Omp19 and its C-terminal domain.** **(A**) Thermal transitions assessed by CD at 230 nm of U-Omp19 and U-Omp19_(60-159)_ in the absence **(left panel)** or in the presence **(right panel)** of a reducing agent. The midpoints of the thermal transitions (Tm) are indicated in the table on the right. **(B)** Superposition of Far-UV CD spectra of the native or chemically denatured (9M urea) states of U-Omp19 and U-Omp19_(60-159)._ Urea unfolding **(C)** and refolding **(D)** transitions followed by Far-UV CD of U-Omp19 and U-Omp19_(60-159)_. The results are expressed as fraction of protein unfolded.

**Fig. S5.**

**
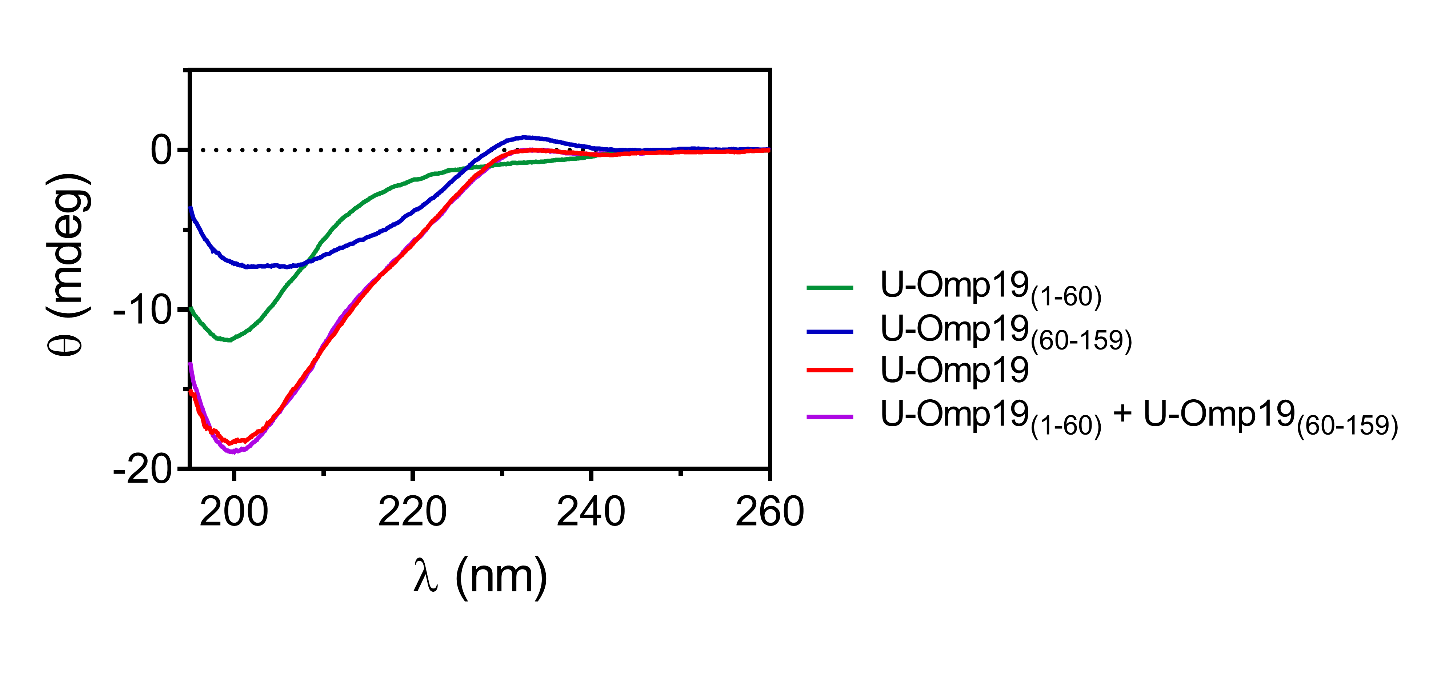
**

**Fig. S5.** Analysis of U-Omp19 domains interaction by Far-UV CD. Individual Far-UV CD spectrum of U-Omp19_(60-159)_ (blue), U-Omp19_(1-60)_ (green) and U-Omp19 (red). The arithmetic sum of the individual spectra of U-Omp19_(60-159)_ and U-Omp19_(1-60)_ is shown in purple.

**Fig. S6**

**
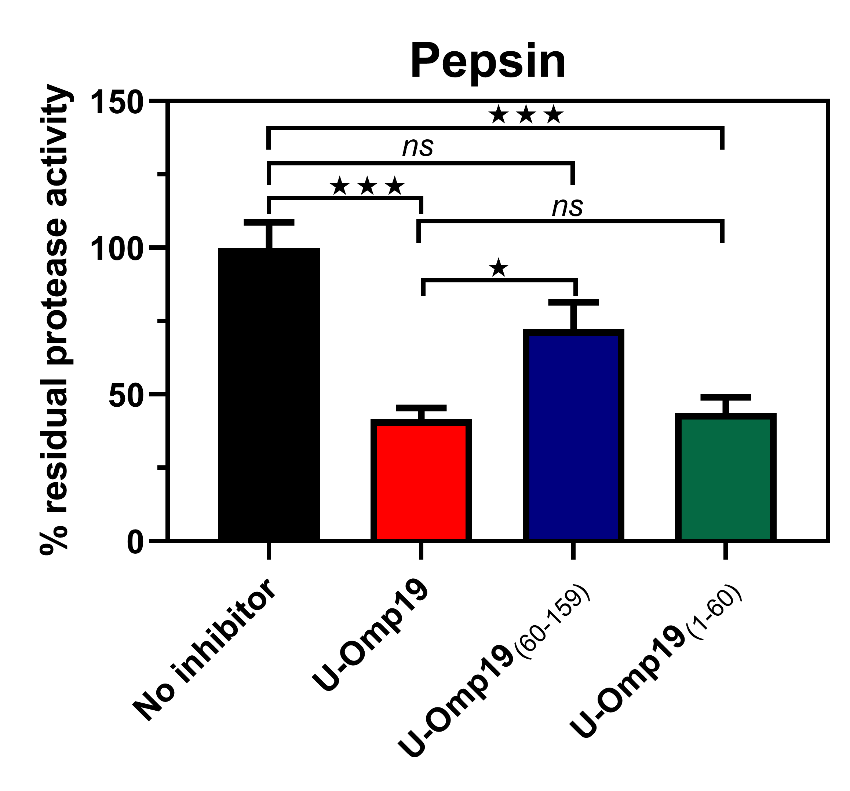
**

**Fig.S6. Protease inhibitor activity of U-Omp19 against pepsin is retained in the N terminal domain of U-Omp19.** Pepsin was incubated for 1 h with buffer (No inhibitor), U-Omp19, U-Omp19_(60-159)_ or U-Omp19_(1-60)_. The residual protease activity was determined after addition of Casein-Bodipy protease fluorogenic substrate and is expressed as the percentage of protease activity remaining when compared to the “No inhibitor” condition (100% of activity). Data were analyzed by one-way ANOVA followed by Bonferroni’s test, ns P > 0.05, ^★^ P < 0.05; ^★★★^ P < 0.001 *vs*. “No inhibitor” or U-Omp19 condition.

**Fig. S7**


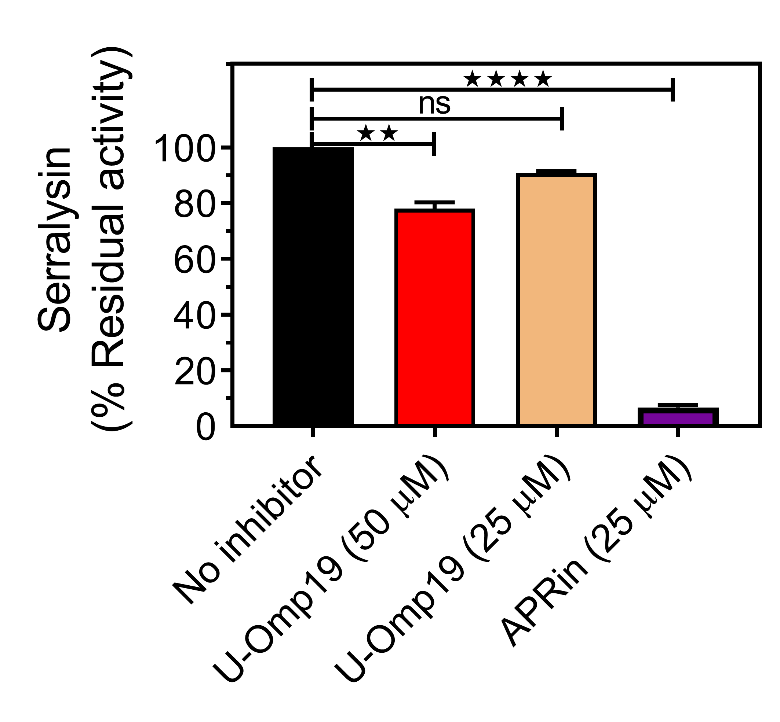


**Fig. S7. Comparison of inhibitor potency of U-Omp19 and APRin against the Serralysin protease.** Serralysin was incubated for 1 h with U-Omp19 (50 µM or 25 µM), APRrin (50 µM) or buffer (No inhibitor). The residual protease activity was determined after addition of a fluorogenic substrate and is expressed as the percentage of protease activity remaining when compared to the “No inhibitor” condition (100% of activity). Data were analyzed by one-way ANOVA followed by Bonferroni’s test, ns P >0.05; ^★★^ P < 0.01; ^★★★★^ P < 0.0001 *vs.* “No inhibitor” condition.
